# What do cost-effective health behaviour-change interventions contain? A comparison of six domains

**DOI:** 10.1371/journal.pone.0213983

**Published:** 2019-04-17

**Authors:** Emma Beard, Robert West, Fabiana Lorencatto, Ben Gardner, Susan Michie, Lesley Owens, Lion Shahab

**Affiliations:** 1 Department of Behavioural Science and Health, University College London, London, United Kingdom; 2 Department of Clinical, Educational and Health Psychology, Centre for Outcomes Research and Effectiveness, University College London, London, United Kingdom; 3 Department of Psychology, Institute of Psychiatry, Psychology and Neuroscience, King’s College London, London, United Kingdom; 4 National Institute for Health and Care Excellence, NICE, London, United Kingdom; University of Nebraska Medical Center, UNITED STATES

## Abstract

**Objectives:**

To help implement behaviour change interventions (BCIs) in practice it is important to be able to characterize their key components. This study compared broad features of cost-effective BCIs that addressed smoking, diet, physical activity, alcohol and sexual health. It also assessed the association of these with the magnitude of the cost-effectiveness estimates.

**Methods:**

A content analysis of 79 interventions based on 338 intervention descriptions was conducted, using the Behaviour Change Wheel (BCW) to classify intervention content in terms of intervention functions, and the BCT taxonomy to identify and categorise component Behaviour Change Techniques (BCT). Regression analysis identified the association of these with upper (pessimistic) and lower (optimistic) cost-effectiveness estimates.

**Results:**

The most and least common functions and BCT clusters were education (82.3%) and shaping knowledge (79.7%), and coercion (3.8%) and covert learning (2.5%). Smoking interventions contained the largest ($\overset{-}{M}$ = 12) number of BCTs and were most cost-effective. Several other factors were associated with worse (coercion_function_ β_upper_ = 36551.24; shaping knowledge_BCT_ β_lower_ = 2427.78; comparison of outcomes_BCT_ β_upper_ = 9067.32; repetition and substitution_BCT_ β_upper_ = 7172.47) and better (modelling_function_ β_lower_ = -2905.3; environmental restructuring_function_ β_upper_ = -8646.28; reward and threat_BCT_ β_upper_ = -5577.59) cost-effectiveness (p<0.05).

**Discussion:**

Cost-effective BCIs rely heavily on education with smoking interventions exhibiting the most comprehensive range of BCTs. Providing an example to aspire to, restructuring the environment and rewarding positive behaviour may be associated with greater cost-effectiveness.

## Introduction

Physical inactivity, smoking, excessive alcohol consumption, unprotected sex and poor diet cost the National Health Service (NHS) in England more than £14 billion per year [1–4]. In the US, smoking alone accounts for 6–8% of personal health expenditure and obesity for 10% of all medical costs [5, 6]. Poor health behaviours also adversely affect the local economy in terms of work productivity, sick leave, and need for social-care [7–9]. Developing behaviour change interventions (BCIs) to address this has become a key objective of public health over the last few decades. BCIs typically involve coordinated sets of activities designed to change specified behaviour patterns. There is good evidence for the effectiveness of some BCIs in some contexts, including the provision of behavioural support for smoking cessation [10], brief advice in primary care for excessive alcohol consumption [11], school based programmes to raise physical activity levels [12], interactive digital interventions for sexual health promotion [13], and behavioural support to reduce calorie intake [14]. These BCIs have also been found to be cost-effective in yielding quality-adjusted life years [15–17].

To implement these interventions in practice it is important to be able to characterize their key components. Also, different types of components may be more useful for some behavioural targets than others. This paper presents a first attempt to specify intervention content and compare this across different behavioural domains using a reliable, theory-based coding system. It focuses specifically on interventions found to be cost-effective [18]. This is because of the common challenge of translating interventions that have been found to be cost-effective in Randomised Controlled Trials (RCTs) to routine practice [19]. Many factors may contribute to this problem [20], but an inadequate specification of the key components of the intervention likely plays a role. In addition, it is often not possible to replicate an intervention precisely, and so it is important to have an understanding of what may be its essential functions so that intervention adaptations can be made without losing these functions [21]. A secondary aim was to identify the association of these key components with measures of cost-effectiveness. This will give some indication as to the degree of cost-effectiveness of different features. To our knowledge this is the first paper to conduct this type of analysis.

BCIs may be characterised in terms of both ‘content’ and ‘delivery’. Content refers to what may be thought of as the active ingredients of the intervention (akin to the chemical composition of a pharmaceutical product), while delivery refers to the manner in which this is applied (akin to the dosing regimen of the pharmaceutical product). The complexity of BCIs means that it is not possible to capture every aspect of content and delivery, but it is possible to capture some key features using coding systems that can be applied with an acceptable degree of reliability [21]. In particular, BCI content can be characterised in terms of a set of ‘intervention functions’, which capture ways in which an intervention can change behaviour: education, persuasion, incentivisation, coercion, training, restriction, environmental restructuring, modelling, and enablement (Table 1).

**Table 1:** Intervention functions.

*Intervention type*	*Definition*	Examples
Education	Increasing knowledge or understanding	Providing information to promote healthy eating
Persuasion	Using communication to induce positive or negative feelings or stimulate action	Using imagery to motivate increases in physical activity
Incentivisation	Creating expectation of reward	Using prize draws to induce attempts to stop smoking
Coercion	Creating expectation of punishment or cost	Raising the financial cost to reduce excessive alcohol consumption
Training	Imparting skills	Advanced driver training to increase safe driving
Restriction	Using rules to reduce the opportunity to engage in the target behaviour (or to increase the target behaviour by reducing the opportunity to engage in competing behaviours)	Prohibiting sales of solvents to people under 18 to reduce use for intoxication
Environmental restructuring	Changing the physical or social context	Providing on-screen prompts for GPs to ask about smoking behaviour
Modelling	Providing an example for people to aspire to or imitate	Using TV drama scenes involving safe-sex practices to increase condom use
Enablement	Increasing means/reducing barriers to increase capability or opportunity^1^	Behavioural support for smoking cessation, medication for cognitive deficits, surgery to reduce obesity, prostheses to promote physical activity

Note: Adapted from Michie et al (2011)

These functions form part of a framework for developing interventions called ‘The Behaviour Change Wheel’ (BCW). The BCW is a synthesis of 19 behaviour change frameworks that draw on a wide range of disciplines and approaches and has been used in a variety of contexts [22–26]. In brief, the BCW is a behavioural system, the hub of which specifies that for behaviour change to occur one needs three conditions: capability, opportunity and motivation (COM-B). Around this hub, nine intervention functions are positioned which aim to address deficits in one or more of these conditions. These intervention functions can then be implemented in an intervention using one of 93 proposed Behaviour Change Techniques (BCTs) [21, 27]. BCTs represent observable and irreducible intervention components that serve to perform one or more of these functions that is, a technique is proposed to be an ‘active ingredient’ (e.g., feedback, self-monitoring, reinforcement) [28]. For example, motivation can be increased through the function ‘persuasion’ and the application of the BCT ‘salience of consequences’, which uses methods to emphasize the consequences of changing the behaviour. This might involve showing people hard hitting images of the consequences of smoking, such as diseased lungs. These coding frameworks have been demonstrated to be able to be used to reliably code descriptions of interventions, and they appear to cover most if not all the BCIs that have been evaluated in RCTs to date [21, 27, 29–35].

It is possible that different behaviours require different approaches, and so these essential functions may differ across behavioural domains. If there are systematic differences, this may provide clues as to the mechanisms of action that commonly need to be targeted in each case. It is possible that effective components of interventions targeting addictive behaviours such as smoking and alcohol consumption may have less of an impact on sexual health, physical activity or diet interventions [36, 37]. For example, pharmacological aids are commonly used for smoking cessation and alcohol dependency, to a lesser extent for weight loss, and infrequently for encouraging exercise [38, 39]. If on the other hand, similar features are found across behavioural domains this may support the use of multiple behaviour change interventions [40]. Identifying whether disparities exist across health behaviours can also be helpful in identifying whether implicit theoretical assumptions exist regarding causes of behaviour. Traditionally, the biomedical model has been used in the treatment of addiction, with recognition only recently of the role of psychological and social factors [37].

The current study aimed to:

1. Characterise BCIs according to their intervention functions [27, 31] and BCTs taxonomy [21]. In order to help understand the findings, this study also classified the interventions according to a range of contextual factors such as setting and intensity.
2. Compare the intervention functions and BCTs used to address smoking, diet, physical activity, alcohol, sexual health, and multiple health behaviours.
3. Assess the association between the features and content of BCIs (in terms of functions and BCTs) with estimates of cost-effectiveness.

## Methods

### Stage 1: Identification and retrieval of source material

Nineteen National Institute for Health and Care Excellence (NICE) guidance documents which included economic modelling and cost-effectiveness reviews and which assessed behaviour change in at least one of six behavioural domains (smoking, diet, physical activity, alcohol, sexual health, or multiple health behaviours) were identified in November/December 2012 through a systematic search conducted by NICE (see Table 2). These reports were searched for interventions which focused on individuals aged 16 years and older and which showed evidence of being cost-effective. A total of 79 interventions were considered to be cost-effective; of which, estimates could be calculated for 72 (see Table 2 and S1 Fig).

**Table 2:** NICE guidance.

Health-Behaviour	Guidance	Cost-effective Intervention Type
Smoking	PH1 Brief interventions and referral for smoking cessation [41]	GP opportunistic advice
		GP opportunistic advice + Nicotine Replacement Therapy (NRT)
		GP opportunistic advice + referral to telephone helpline
		GP opportunistic advice + self-help materials
		Nurse-led brief intervention in primary care
	PH5 Workplace interventions to promote smoking cessation [42]	Nurse-led brief intervention in hospital setting
		Brief advice + self-help material + NRT
		Brief advice + self-help material+ NRT + specialist clinic
	PH10 Smoking cessation services [43]	Less intensive counselling + bupropion
		More intensive counselling + bupropion
		NRT 5 weeks + 5 clinic visits
		NRT 5 weeks + 5 group visits
		NRT 5 weeks
		NRT 5 weeks + 5 pharmacy consultations
	PH15 Identifying and supporting people most at risk of dying prematurely [44]	NRT 5 weeks + Pharmacy consultations
		NRT 5 weeks + 5 pharmacy consultations + 5 clinic visits
		Client-centered social marketing interventions
		Workplace intervention to improve access
		Brief advice for pregnant smokers
		Proactive telephone support for pregnant smokers
		NRT prescription incentives
		NHS Stop Smoking Services identifying and reach smokers
		Pediatric unit identifying and reaching smokers
		Pharmacy-based recruitment
		Recruiting smokers from community
		Social marketing to deliver client centered approaches to smoking cessation
		Free mobile phones for use in smoking cessation counselling
		Cervical screening recruitment
		Nurse run clinics
		Proactive telephone counselling
		Quit and win recruitment
		Identifying smokers through other means
		Dentist-based interventions to improve access
	PH26 Quitting smoking in pregnancy and following childbirth [45]	Drop-in/rolling community based sessions to improve access
		Pharmacy-based interventions to improve access
		Free NRT incentives
		Workplace smoking cessation and incentives
		Cognitive behaviour strategies
		Stages of change
		Feedback
		Rewards
		Pharmacotherapy
Diet	PH11 Maternal and child nutrition [46]	Peer support
	PH27 Weight management before, during and after pregnancy [45]	Folic acid supplement
		Women, infants and children (WIC) programme
		Diet
		Behavioural treatment
		Exercise
		Diet and exercise
Physical activity	PH2 Four commonly used methods to increase physical activity [47]	Brief interventions
		Exercise referral
	PH8 Physical activity and the environment [48]	Urban planning and design
		Transport
		Building design
	PH13 Promoting physical activity in the workplace [49]	Physical activity counselling
	PH17 Promoting physical activity for children and young people [50]	Physical activity programme
		Family-based behavioural treatment
Alcohol	PH7 School-based interventions on alcohol [51]	Stars for families brief intervention
		The School Health and Harm Reduction Programme (SHAHRP)
	PH24 Alcohol use disorders: preventing harmful drinking [52]	Lion’s Quest ‘Skills for adolescence’ programme
		Brief intervention and screening in Primary Care
		Brief intervention and screening in Emergency Care
		Pricing and price-based promotion policies
		Reduction in outlet density
		Reduction in licensing hours
		Advertising ban
		Reinforcing driver/server laws
Sexual health	PH3 Prevention of sexually transmitted infections and under 18 conceptions [51]	Accelerated Partner Therapy
		Patient referral at GP
		Brief counselling
		Enhanced/intensive counselling
		Tailored skills session
		Behaviour skills counselling
	PH33 increasing the uptake of HIV testing among black Africans in England [53]	No interventions included in the cost-effectiveness review
	PH34 Increasing the uptake of HIV testing among men who have sex with men [54]	Peer education and recruitment
Multiple health behaviours	PH6 Behaviour change [55]	Multiple component CHD prevention programme
	PH25 Prevention of cardiovascular disease [56]	Population-wide multifactor intervention
		Multi-component intervention
	PH35 Preventing type 2 diabetes [57]	Dietary, nutritional and educational
		Multi-component
		Large-scale, region-wide multi-component

Cost-effectiveness was determined using cost-utility analysis. This considers someone’s quality of life and the length of life they will gain as a result of an intervention. The health benefits are expressed as quality-adjusted life years (QALYs). An intervention was classified as cost-effective if its incremental cost-effectiveness ratio (ICER), which is the difference in cost between two possible interventions, divided by the difference in their effect, was below the NICE threshold of £20,000 per quality-adjusted life year (QALY) [58]. Cost-effectiveness estimates were derived directly or calculated from figures in the reports and, where necessary, converted into GBP at time of original analysis. Both lower and upper limits of estimates were recorded.

After identification of cost-effective interventions in economic reports, the sources of effectiveness estimates used in economic analyses of these interventions were identified. These could either be publications reporting primary data, or summaries located in systematic reviews/meta-analyses. A total of 115 relevant source documents were initially identified from economic analyses. In cases where insufficient detail was provided on intervention content in systematic reviews/meta-analyses, original studies were retrieved, resulting in a total of 338 papers/reviews being reviewed (66 reviews and 272 original papers; see S1 Material).

### Stage 2: Characterisation of interventions

The content of interventions was characterised using two methods. The first identified their intervention functions as defined in the BCW framework [31] (see Table 1). Interventions were also coded using a taxonomy of 93 BCTs [BCT Taxonomy v1 [21]], divided into 16 clusters derived from hierarchical cluster analysis (see [21] for more details).

Following Michie et al’s [21] guidelines, BCTs were coded only where coders believed that there was unequivocal evidence of their inclusion in a given intervention. All articles were coded by EB, with a subset of articles (n = 66, 20%) coded in batches by BG, with disagreements resolved through discussion after each batch. Agreement was 97%, with a mean Cohen’s Kappa of 0.74 (95%CI 0.67 to 0.82), indicating good inter-rater reliability [59].

Interventions were also categorized in terms of a range of factors relating to their context and delivery: intervention level (e.g. individual vs. population), delivery agent type (e.g. nurses vs. physicians), and intensity (e.g. high vs. low) [60–62] (see S2 Table).

## Analysis

All data were extracted into a data extraction form and then transferred into IBM SPSS v.20. Interventions were split into six categories: alcohol, diet, smoking, physical activity, sexual-health interventions, and interventions targeting multiple health behaviours. Differences according to intervention characteristics were analysed using t-tests or one-way ANOVAs and Chi-square (χ^2^) or Fisher Exact tests for continuous and categorical variables, respectively. The Tukey correction was applied in post hoc analyses.

The factors associated with cost-effectiveness were then assessed using generalised linear modelling specifying the Gaussian family. Only the intervention features, functions and BCT clusters were considered due to sample size. Unadjusted and stepwise adjusted models are reported for all interventions combined for which cost-effectiveness estimates were available (n = 72). Stepwise methods were used to select the most relevant variables for the adjusted analysis based on the Akaike information criterion (AIC). Associations were assessed with both lower (most optimistic) and upper (most pessimistic) limits of cost-effectiveness estimates taken directly from the reports. In cases where only a single cost-effectiveness estimate was recorded this was included as both the lower and upper limit.

## Results

### Broad characterization of interventions

#### Overall

The majority of interventions were classified as being of high intensity, were set in primary care or the community and were delivered by health professionals (Table 3). They also mostly targeted individuals from the general population. Incentives were used in 15.2% of interventions and pharmacological support in 34.2%.

**Table 3:** Intervention characteristics by health behavior.

	All	Smoking (n = 41)^1^	Diet (n = 7) ^2^	Physical activity (n = 8) ^3^	Alcohol (n = 10) ^4^	Sexual health (n = 7) ^5^	Multiple health behaviours (n = 6) ^6^	*p*
	*% (N)*							
Intervention intensity								0.456
*Low*	36.7 (29)	36.6 (15)	14.3 (1)	62.5 (5)	50.0 (5)	28.6 (2)	16.7 (1)	
*Medium*	16.5 (29)	17.1 (7)	0 (0)	12.5 (1)	20.0 (2)	28.6 (2)	16.7 (1)	
*High*	46.8 (37)	46.3 (19)	85.7 (6)	25.0 (2)	30.0 (3)	42.9 (3)	66.7 (4)	
Setting		^a^^,^^b^	^a^	^a^^,^^b^	^a^^,^^b^	^b^	^a^^,^^b^	0.028
*Primary care*	34.2 (27)	41.5 (17)	0 (0)	12.5 (1)	10.0 (1)	85.7 (6)	33.3 (2)	
*Secondary care*	2.5 (2)	2.4 (1)	0 (0)	0 (0)	10.0 (1)	0 (0)	0 (0)	
*Community*	26.8 (21)	22.0 (9)	71.4 (5)	37.5 (3)	10.0 (1)	14.3 (1)	33.3 (2)	
*Workplace*	13.9 (11)	14.6 (6)	0 (0)	37.5 (3)	20.0 (2)	0 (0)	0 (0)	
*Other*^≠^	22.8 (18)	19.5 (8)	28.6 (2)	12.5 (1)	50.0 (5)	0 (0)	33.3 (2)	
Delivery mode		^a^	^a^^,^^b^	^a^^,^^b^	^b^	^a^^,^^b^	^a^^,^^b^	0.002
*Physician*	12.7 (10)	14.6 (6)	0 (0)	0 (0)	20.0 (2)	14.3 (1)	16.7 (1)	
*HP*	48.1 (38)	53.7 (22)	71.5 (5)	37.5 (3)	10.0 (1)	71.4 (5)	33.3 (2)	
*Media*	5.1 (4)	9.8 (4)	0 (0)	0 (0)	0 (0)	0 (0)	0 (0)	
*Mix*	13.9 (11)	17.1 (7)	0 (0)	12.5 (1)	0 (0)	0 (0)	50.0 (3)	
*Other*^±^	20.3 (16)	4.9 (2)	28.6 (2)	50.0 (4)	70.0 (7)	14.3 (1)	0 (0)	
Target Level		^a^	^a^^,^^b^	^a^^,^^b^	^b^	^a^^,^^b^	^b^	0.008
*Individual*	69.6 (55)	82.9 (34)	71.4 (4)	62.5 (5)	30.0 (3)	100 (7)	16.7 (1)	
*Groups*	12.7 (10)	9.8 (4)	14.3 (1)	12.5 (1)	20.0 (2)	0 (0)	33.3 (2)	
*Population*	17.7 (14)	7.3 (3)	14.3 (1)	25.0 (2)	50.0 (5)	0 (0)	50.0 (3)	
Population								0.063
*General*	68.4 (54)	63.4 (26)	42.9 (3)	100 (8)	70.0 (7)	100 (7)	50.0 (3)	
*Vulnerable*	31.6 (25)	36.6 (15)	57.1 (4)	0 (0)	30.0 (3)	0 (0)	50.0 (3)	
Supporting Material								0.504
*None*	54.4 (43)	46.3 (19)	85.7 (6)	62.5 (5)	80.0 (8)	57.1 (4)	16.7 (1)	
*Self-help*	32.9 (26)	34.1 (14)	14.3 (1)	25.0 (2)	20.0 (2)	42.9 (3)	66.7 (4)	
*Electronic*	3.8 (3)	7.3 (3)	0 (0)	0 (0)	0 (0)	0 (0)	0 (0)	
*Mix*	8.9 (7)	12.2 (5)	0 (0)	12.5 (1)	0 (0)	0 (0)	16.7 (1)	
Pharmacological support	34.2 (27)	58.5 (24)^a^	14.3 (1)^a^^,^^b^	0 (0)^b^	0 (0)^b^	14.3 (1)^a^^,^^b^	16.7 (1)^a^^,^^b^	<0.001
Social marketing	12.7 (1)	12.2 (5)	0 (0)	12.5 (1)	10.0 (1)	0 (0)	50.0 (3)	0.085
Incentives	15.2 (12)	14.6 (6)	0 (0)	25.0 (2)	20.0 (2)	14.3 (1)	16.7 (1)	0.841

Note: *HP* Health professional (nurse, pharmacist, psychologist etc)

^1^From 6 economic reports

^2^From 4 economic reports

^3^From 4 economic reports

^4^From 2 economic reports

^5^From 3 economic reports

^6^From 4 economic reports

^≠^This refers to state/policy level interventions (e.g., changes in legislation or physical infrastructure) or interventions in non-specific settings (e.g., online/phone interventions)

^±^This refers to delivery by peers, teachers, researchers or the state

^a,b^ Different letters indicate significant difference at p<0.05 between categories in that row, shared letters indicate no differences (Tukey-corrected)

#### By behavioural domain

The broad intervention features used varied according to behavioural domain. While diet interventions were mostly set in the community, interventions to improve sexual health were predominantly based in primary care. Post-hoc analysis also showed that smoking interventions were generally delivered by health professionals or physicians. Interventions for alcohol consumption and those targeting multiple health behaviours were more often population-wide than those for other target behaviours, which mostly focused on individuals or groups. Smoking cessation interventions were also more likely to involve pharmacological support than other interventions.

### Intervention functions

#### Overall

Overall, the most common functions, identified in over two thirds of interventions, were education (82.3%), and enablement (75.9%) (Table 4). Only 3.8% of interventions used coercion and 7.6% incentivisation.

**Table 4:** Intervention functions by health behavior.

	All (N = 79)	Smoking (N = 41)^1^	Diet (N = 7)^2^	Physical Activity (N = 8)^3^	Alcohol (N = 10)^4^	Sexual Health (N = 7)^5^	Multiple health behaviours (N = 6)^6^	*p*
	*% (N)*							
Education	82.3 (65)	90.2 (37)^a^	85.7 (6)^a^^,^^b^	62.5 (5)^a^^,^^b^	50.0 (5)^b^	85.7 (6)^a^^,^^b^	100 (6)^a^^,^^b^	0.040
Enablement	75.9 (60)	78.0 (32)^a^	71.4 (5)^a^^,^^b^	100 (8)^a^	30.0 (3)^b^	85.7 (6)^a^^,^^b^	100 (6)^a^^,^^b^	0.003
Training	57.0 (45)	53.7 (22)^a^	85.7 (6)^a^	75.0 (6)^a^	0 (0)^b^	85.7 (6)^a^	83.3 (5)^a^	<0.001
Persuasion	55.7 (44)	73.2 (30)^a^	14.3 (1)^a^^,^^b^	37.5 (3)^a^^,^^b^	20.0 (2)^b^	71.4 (5)^a^^,^^b^	50.0 (3)^a^^,^^b^	0.003
Environmental restructuring	21.5 (17)	4.9 (2)^a^	42.9 (3)^a^^,^^b^	50.0 (4)^b^	50.0 (5)^b^	0 (0)^a^^,^^b^	50.0 (3)^a^^,^^b^	0.001
Modelling	15.2 (12)	4.9 (2)	42.9 (3)	25.0 (2)	0 (0)	28.6 (2)	50.0 (3)	0.007
Restriction	12.6 (10)	0 (0)^a^	28.6 (2)^a^^,^^b^	0 (0)^a^^,^^b^	60.0 (6)^b^	28.6 (2)^a^^,^^b^	0 (0)^a^^,^^b^	<0.001
Incentivisation	7.6 (6)	14.6 (6)	0 (0)	0 (0)	0 (0)	0 (0)	0 (0)	0.304
Coercion	3.8 (3)	0 (0)	0 (0)	0 (0)	10.0 (1)	28.6 (2)	0 (0)	0.059

Note:

^1^From 6 reports

^2^From 4 reports

^3^From 4 economic reports

^4^From 2 economic reports

^5^From 3 economic reports

^6^From 4 economic reports

^a,b^ Different letters indicate significant difference at p<0.05 between categories in that row, shared letters indicate no differences (Tukey-corrected)

#### By behavioural domain

Intervention functions also differed significantly according to the health behaviour targeted. In contrast to other types of interventions, alcohol interventions had a weaker focus on education, enablement and training and a stronger focus on restrictions (Table 4). While alcohol and diet interventions also had less of a focus on persuasion, the use of environmental restructuring was particularly uncommon in smoking cessation and sexual health interventions. There were no differences between interventions in terms of incentivisation, coercion or modelling in post-hoc analyses.

### Identification of BCT clusters

#### Overall

Around three-quarters of BCIs included the BCT cluster ‘shaping knowledge’ (79.7%), which includes the BCTs reattribution, antecedents, behavioural experiments and instruction on how to perform the behaviour, and the cluster ‘goals and planning’ (73.4%, Table 5). More than 60% of the interventions also included ‘social support’ and ‘antecedents’ while only 3.8% included ‘scheduled consequences’, and 2.5% ‘covert learning’.

**Table 5:** Behaviour Change Technique clusters by health behavior.

	All (N = 79)	Smoking (N = 41)^1^	Diet (N = 7)^2^	Physical Activity (N = 8)^3^	Alcohol (N = 10)^4^	Sexual Health (N = 7)^5^	Multiple health behaviours (N = 6)^6^	*p*
	*% (N)*							
Shaping knowledge (BCT36-39)	79.7 (63)	90.2 (37)^a^	100 (7)^a^^,^^b^	62.5 (5)^a^^,^^b^	30.0 (3)^b^	85.7 (6)^a^^,^^b^	83.3 (5)^a^^,^^b^	0.002
Goals and planning (BCT65-73)	73.4 (58)	85.4 (35)^a^	28.6 (2)^b^	75.0 (6)^a^^,^^b^	50.0 (5)^a^^,^^b^	71.4 (5)^a^^,^^b^	83.3 (5)^a^^,^^b^	0.030
Social support (BCT1-3)	68.4 (54)	80.5 (33)	28.6 (2)	75.0 (6)	50.0 (5)	42.9 (3)	83.3 (5)	0.036
Antecedents (BCT30-35)	63.3 (50)	58.5 (24)	100 (7)	87.5 (7)	50.0 (5)	42.9 (3)	66.7 (4)	0.130
Natural consequences (BCT82-87)	58.2 (46)	68.3 (28)	14.3 (1)	62.5 (5)	50.0 (5)	28.6 (2)	83.3 (5)	0.032
Comparison of outcomes (BCT75-BCT78)	51.9 (41)	63.4 (26)^a^	0.0 (0)^b^	37.5 (3)^a^^,^^b^	50.0 (5)^a^^,^^b^	57.1 (4)^a^^,^^b^	50.0 (5)^a^^,^^b^	0.021
Feedback and monitoring (BCT8-14)	50.6 (40)	51.2 (21)	42.9 (3)	25.0 (2)	30.0 (3)	71.4 (5)	100 (6)	0.051
Regulation (BCT4-7)	44.3 (35)	75.6 (31)^a^	0 (0)^b^	0 (0)^b^	20.0 (2)^b^	14.3 (1)^b^	16.7 (1)^a^^,^^b^	<0.001
Comparison of behaviour (BCT88-90)	35.4 (28)	36.6 (15)	14.3 (1)	37.5 (3)	30.0 (3)	42.9 (3)	50.0 (3)	0.812
Self-beliefs (BCT40-43)	34.2 (27)	43.9 (18)	14.3 (1)	25.0 (2)	30.0 (3)	42.9 (3)	0 (0)	0.245
Reward and threat (BCT54-64)	32.9 (26)	41.5 (17)	0 (0)	50.0 (4)	10.0 (1)	28.6 (2)	33.3 (2)	0.141
Repetition and substitution (BCT23-29)	31.6 (25)	41.5 (17)	14.3 (1)	12.5 (1)	20.0 (2)	42.9 (3)	16.7 (1)	0.325
Associations (BCT15-22)	29.1 (23)	34.1 (14)	14.3 (1)	12.5 (1)	40.0 (4)	0 (0)	50.0 (3)	0.218
Identity (BCT77-81)	17.7 (14)	12.2 (5)	0 (0)	12.5 (1)	40.0 (4)	42.9 (3)	16.7 (1)	0.111
Scheduled consequences (BCT44-53)	3.8 (3)	7.3 (3)	0 (0)	0 (0)	0 (0)	0 (0)	0 (0)	0.717
Covert learning (BCT91-93)	2.5 (2)	4.9 (2)	0 (0)	0 (0)	0 (0)	0 (0)	0 (0)	0.863

Note:

^a,b^ Different letters indicate significant difference at p<0.05 between categories in that row, shared letters indicate no differences (Tukey-corrected)

^1^From 6 reports

^2^From 4 reports

^3^From 4 economic reports

^4^From 2 economic reports

^5^From 3 economic reports

^6^From 4 economic reports

#### By behavioural domain

The prevalence of clusters differed among the behavioural domains (Table 5). All of the diet interventions included shaping knowledge and antecedents, while fewer than 50% of alcohol interventions included these two clusters. Goals and planning and social-support were most prevalent in smoking and multiple health behaviour interventions and least prevalent in diet interventions which also did not feature comparison of outcomes, which includes the BCTs persuasive source, pros and cons and comparative imagining of future outcomes. The BCT cluster regulation was most common in smoking interventions which were also the only interventions that included scheduled consequences and covert learning, this involves the BCTs imaginary punishment and reward and vicarious consequences. All the multiple health behaviour interventions included feedback and monitoring.

### Identification of individual BCTs

#### Overall

The mean number of BCTs identified per BCI was 10 (range 2 to 39). Smoking cessation interventions included the largest number of BCTs on average (mean 11.8, median = 8, mode = 6, range 3 to 39), followed by interventions targeting multiple behaviours (mean = 9.1, median = 8, mode = 7, range 6 to 14),physical activity interventions (mean = 8.4, median = 7.5, mode = 4, range 4 to 14), alcohol interventions (mean = 7.7, median = 5.5, mode = 3, range 2 to 21) and interventions to improve sexual health (mean = 7.6, median = 5, mode = 5, range 2 to 15). Diet interventions included the smallest number of BCTs (mean = 4.7, median = 5, mode = 5, range 2 to 8).

**Fig 1:**
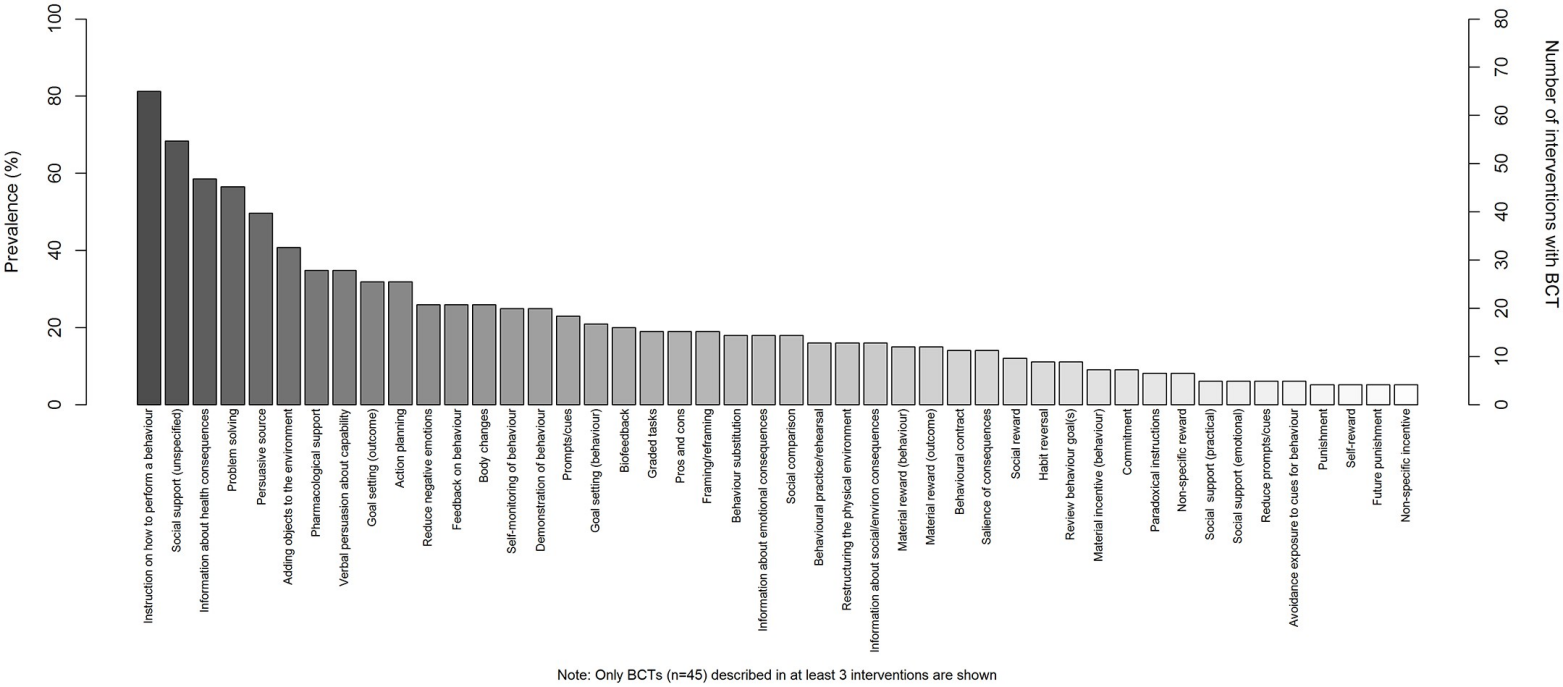
Prevalence of individual BCTs across all interventions.

A total of 45 BCTs were identified in at least three BCIs (see Fig 1). Instructions on how to perform a behaviour, social support (unspecified), information about health consequences and problem solving, which involves analyzing factors influencing the behaviour and generating strategies that include overcoming barriers and/or increasing facilitators, were included in over half of all the BCIs. A further four BCTs were coded in at least two interventions [mental rehearsal of successful performance, self-incentive, information about other’s approval, imaginary punishment] and a further nine BCTs were included in at least one intervention [addition of self-monitoring of outcome(s) of behaviour, monitoring outcome(s) of behaviour by others without feedback, remove aversive stimulus, satiation, restructuring the social environment, distraction, information about antecedents, incompatible beliefs, identity associated with changed behaviour, anticipated regret]. The other 34 BCTs were not identified in any BCI.

#### By behavioural domain

A total of 55 distinct BCTs were identified in the smoking cessation interventions. The most prevalent BCT (recorded in 90% of smoking cessation interventions) was the inclusion of instructions on how to perform a behaviour, for example, advise the person how to use smoking cessation medication correctly (see S1 Table). Thirteen BCTs were found in the dietary interventions. Instructions on how to perform a behaviour was the most common BCT. Physical activity interventions included 29 BCTs. The most common BCT was discussion of body changes, which involves altering body structure, functioning or support directly to facilitate behaviour change. Thirty BCTs were identified in effective alcohol interventions, and 23 in interventions to improve sexual health. All sexual-health interventions included instructions on how to perform a behaviour. Finally, in the interventions targeting multiple behaviours, 23 BCTs were identified.

### Factors associated with cost-effectiveness

In bivariate analyses, sexual health interventions were found to be less cost-effective than smoking cessation interventions (β_lower_ = 7422.54, p<0.001 and β_upper_ = 25190.52, p<0.001), while interventions implemented in nonstandard settings were more cost effective than those set in primary care (β_upper_ = -8799.06, p = 0.036). Several functions and BCT clusters were also associated with lower cost-effectiveness: restriction (β_lower_ = 3322.8, p = 0.008 and β_upper_ = 13144.1, p = 0.004), coercion (β_lower_ = 9947.6, p<0.001 and β_upper_ = 45665.9, p<0.001) and identity (β_lower_ = 2226.4, p = 0.044 and β_upper_ = 9882.2, p = 0.014) (see Table 6).

**Table 6:** Factors associated with upper and lower cost-effectiveness estimates.

	Unadjusted						Adjusted stepwise model					
	Lower cost-effectiveness estimate			Upper cost-effectiveness estimate			Lower cost-effectiveness estimate			Upper cost-effectiveness estimate		
	Β	95%CI	p*	β	95%CI	p*	Β	95%CI	P	β	95%CI	p*
***Intervention characteristics***												
**Health Behaviour**												
Smoking	Ref			Ref			Ref			Ref		
Diet	2509.26	-22.93 to 5041.45	0.056	7600.81	-2020.84 to 17222.45	0.126	2590.19	-126.86 to 5307.23	0.067	14683.11	5812.71 to 23553.5	0.002*
Physical activity	-784.17	-3177.36 to 1609.02	0.523	-734.23	-9827.73 to 8359.26	0.875	2739.99	376.65 to 5103.34	0.027*	2635.56	-5419.42 to 10690.54	0.524
Alcohol	-75.47	-2259.25 to 2108.31	0.946	343.25	-7954.55 to 8641.05	0.936	1359.56	-1392.55 to 4111.68	0.337	-3646.48	-12762.58 to 5469.61	0.436
Sexual health	7422.54	4890.36 to 9954.73	<0.001*	25190.52	15568.88 to 34812.17	<0.001*	7012.09	4576.56 to 9447.63	<0.001*	7441.94	-1192.03 to 16075.9	0.096
Multiple behaviours	666.16	-2040.27 to 3372.6	0.631	7832.62	-2451.12 to 18116.36	0.140	681.17	-2069.54 to 3431.88	0.629	15431.68	4885.21 to 25978.15	0.006*
**Intervention Intensity**												
Low	Ref			Ref								
Medium	964.03	-1501.96 to 3430.01	0.446	5371.49	-3617.46 to 14360.44	0.245						
High	1348.7	-483.65 to 3181.05	0.153	4683.99	-1995.25 to 11363.23	0.173						
**Setting**												
Primary Care	Ref			Ref								
Secondary Care	2045.97	-3315.03 to 7406.98	0.457	-4027.06	-23484.8 to 15430.68	0.686						
Community	-1221.96	-3350.45 to 906.54	0.264	-6499.32	-14224.69 to 1226.05	0.103						
Workplace	-2219.9	-4836.63 to 396.82	0.101	-9526.7	-19024.08 to -29.32	0.053						
Other	-1946.75	-4172.78 to 279.29	0.091	-8799.06	-16878.44 to -719.67	0.036*						
**Delivery Mode**												
Physician	Ref			Ref			Ref					
HP	-41.6	-2606.86 to 2523.65	0.975	482.79	-9166.62 to 10132.19	0.922	-1945.68	-3991.95 to 100.59	0.067			
Media	-2754.35	-7024.43 to 1515.73	0.210	-5768.45	-21830.67 to 10293.77	0.484	-5562.95	-10529.36 to -596.54	0.032*			
Mix	-1689.36	-4843.02 to 1464.31	0.297	-1022.6	-12885.34 to 10840.14	0.866	-1735.14	-4231.43 to 761.16	0.178			
Unclear/other	-2664.1	-5573.67 to 245.46	0.077	-6116.95	-17061.51 to 4827.61	0.277	-3986.23	-6663.96 to -1308.5	0.005*			
**Target Level**												
Individual	Ref			Ref						Ref		
Groups	-1955.61	-4463.16 to 551.95	0.131	-5118.63	-14444.49 to 4207.23	0.285				-3740.57	-10646.21 to 3165.08	0.293
Population	-1773.71	-3957.21 to 409.8	0.116	-1545.64	-9666.33 to 6575.04	0.710				-8097.95	-18327.16 to 2131.26	0.126
**Population**	-1955.61	-4463.16 to 551.95	0.131	-5118.63	-14444.49 to 4207.23	0.285				-3740.57	-10646.21 to 3165.08	0.293
Vulnerable	Ref			Ref			Ref			Ref		
General	344.3	-1454.2 to 2142.7	0.709	4101.8	-2404.5 to 10608.2	0.220	-1855.58	-3417.62 to -293.53	0.023*	5588.12	256.04 to 10920.2	0.044*
**Supporting Material**												
None	Ref			Ref								
Self-help	-854.13	-2693.75 to 985.5	0.366	2735.1	-4018.94 to 9489.15	0.430						
Electronic	-2056.52	-6478.43 to 2365.39	0.365	-4358.56	-20593.28 to 11876.15	0.600						
Mix	-2115.92	-5133.98 to 902.13	0.174	-4081.12	-15161.69 to 6999.46	0.473						
**Pharmacological support**	-784.9	-2541.4 to 971.5	0.384	-1437.6	-7872.5 to 4997.3	0.663						
**Social marketing**	-1235.9	-3738.6 to 1266.9	0.336	970.6	-8217.5 to 10158.6	0.837	3216.09	139.67 to 6292.52	0.045*	7505.3	-891.46 to 15902.06	0.085
**Incentives**	-1791.5	-4089.5 to 506.5	0.131	-5271.2	-13703.5 to 3161.2	0.224	-3402.56	-5918.78 to -886.35	0.010*	-5728.62	-12419.2 to 961.97	0.099
***Intervention functions***												
Training	1200.8	-468.7 to 2870.3	0.163	4529.3	-1559.1 to 10617.8	0.149				5382.49	-558.2 to 11323.19	0.081
Education	1242.4	-932.6 to 3417.3	0.266	3077.7	-4895.6 to 11051	0.452				-8815.77	-18478.33 to 846.79	0.079
Enablement	626.9	-1327 to 2580.8	0.531	1447.6	-5695.2 to 8590.4	0.692						
Persuasion	1326.1	-333.1 to 2985.3	0.121	3957.6	-2130.5 to 10045.6	0.206	1136.97	-348.12 to 2622.07	0.139			
Environmental restructuring	-1058.9	-3082.4 to 964.6	0.308	-3898.5	-11283.5 to 3486.5	0.304				-8646.28	-15996.95 to -1295.6	0.025*
Incentivisation	-1428.3	-4572.5 to 1715.9	0.376	-4458.2	-15950.5 to 7034.1	0.449	3427.68	-537.99 to 7393.36	0.096			
Restriction	3322.8	916.7 to 5728.8	0.008*	13144.1	4435.1 to 21853.2	0.004*	2225.11	-519.43 to 4969.66	0.117	7320.34	-3304.96 to 17945.64	0.182
Modelling	389.8	-1941.2 to 2720.8	0.744	6184.9	-2216.4 to 14586.2	0.153	-2905.3	-4980.49 to -830.12	0.008*			
Coercion	9947.6	6172.7 to 13722.5	<0.001*	45665.9	33353.9 to 57977.9	<0.001*	6679.31	2731.51 to 10627.12	0.002*	36551.24	22478 to 50624.49	<0.001*
***BCT clusters***												
Shaping knowledge	2030.5	-2.8 to 4063.8	0.054	2256.5	-5330.6 to 9843.7	0.562	2427.78	418.23 to 4437.32	0.021*	-6853.47	-14151.32 to 444.37	0.071
Antecedents	-602	-2333.7 to 1129.8	0.498	1292.1	-5041.2 to 7625.5	0.690						
Regulation	-571.2	-2251.8 to 1109.4	0.507	-3653.5	-9751 to 2444	0.244						
Social support	-69.5	-1869.6 to 1730.5	0.940	58	-6512.6 to 6628.5	0.986						
Comparison of outcomes	-45.2	-1720.8 to 1630.4	0.958	2545.2	-3544.5 to 8635	0.415	-2026.43	-3667.9 to -384.96	0.019*	9067.32	3581.75 to 14552.89	0.002*
Feedback and monitoring	1626.9	-7.8 to 3261.6	0.055	4772.3	-1246.4 to 10790.9	0.124						
Goals and planning	909.5	-974.7 to 2793.7	0.347	2075.1	-4826.8 to 8977	0.557						
Natural consequences	123.5	-1573.9 to 1820.8	0.887	2470.4	-3701.2 to 8642.1	0.435						
Self-beliefs	501.2	-1260.4 to 2262.8	0.579	3952	-2430.2 to 10334.2	0.229						
Repetition and substitution	1174.4	-606.5 to 2955.3	0.200	4436.9	-2058.5 to 10932.2	0.185				7172.47	1780.36 to 12564.59	0.012*
Comparison of behaviour	121.6	-1628.5 to 1871.6	0.892	5118.9	-1166.5 to 11404.4	0.115						
Associations	-1051.8	-2879.7 to 776.1	0.263	-1055.7	-7778.4 to 5666.9	0.759						
Reward and threat	-8.1	-1789.8 to 1773.6	0.993	-2515.9	-8995 to 3963.2	0.449				-5577.59	-10607.58 to -547.6	0.034*
Identity	2226.4	91 to 4361.8	0.044*	9882.2	2189.7 to 17574.7	0.014*						
Scheduled consequences	-291.9	-4671.7 to 4087.9	0.896	-2716.8	-18693.4 to 13259.9	0.740						
Covert learning	43.8	-5286 to 5373.5	0.987	-3458.6	-22897.2 to 15979.9	0.728						
***Number of BCTS***	6.19	-106.79 to 119.18	0.915	70.82	-341.32 to 482.96	0.737						

Note:

* indicates significance, based on n = 72 reports which provided cost-utility analyses

BCT = Behaviour Change Techniques

In adjusted stepwise analyses, diet (β_upper_ = 14683.11, p = 0.002), physical activity (β_lower_ = 2739.99, p = 0.027), sexual health (β_lower_ = 7012.09, p<0.001) and multiple health behaviour interventions (β_upper_ = 15431.68, p = 0.006) were found to be less cost-effective than smoking cessation interventions. Those delivered through use of media or nonstandard means were more cost-effective than those delivered by a physician (β_lower_ = -5562.95, p = 0.032 and β_lower_ = -3986.23, p = 0.005). Recruitment of a general population sample was associated with higher cost-effectiveness for the lower cost-effectiveness limit and lower cost-effectiveness for the upper cost-effectiveness limit (βlower = -1855.58, p = 0.023 and βupper = 5588.12, p = 0.044), while use of incentives was associated with higher cost-effectiveness and social-marketing with lower cost-effectiveness (β_lower_ = -3402.56, p = 0.010 and β_lower_ = 3216.09, p = 0.045). Several intervention functions were also associated with lower (coercion β_lower_ = 6679.31, p = 0.002 and β_upper_ = 36551.24, p<0.001) and higher (modelling β_lower_ = -2905.3, p = 0.008; environmental restructuring β_upper_ = -8646.28, p = 0.025) cost-effectiveness. The BCT clusters shaping knowledge (β_lower_ = 2427.78, p = 0.021), comparison of outcomes (β_lower_ = -2026.43, p = 0.019 and β_upper_ = 9067.32, p = 0.002) repetition and substitution (β_upper_ = 7172.47, p = 0.012) were associated with lower cost-effectiveness, while reward and threat (β_upper_ = -5577.59, p = 0.034) with higher cost-effectiveness.

## Discussion

This study compared broad features of cost-effective behaviour change interventions (BCIs) that addressed smoking, diet, physical activity, alcohol and sexual health. It also assessed the association of these with cost-effectiveness estimates among cost-effective interventions.

Most interventions were high intensity, set in primary care and delivered by health-care professionals. Education and enablement were the most commonly used intervention functions while incentivisation and coercion were rarely used. There was large variation across behavioural domains. While education, enablement, persuasion and training were less prominent, restriction was more prominent for alcohol than other behavioural targets. The majority of interventions included around 10% of all the potential BCTs in the taxonomy, with the most common BCT clusters being shaping knowledge and goals and planning. Few studies adopted scheduled consequences or covert learning. There was substantial variability across behavioural domains. For example, the use of pharmacological support, persuasive source, social-support and goal–setting were most prevalent in smoking cessation interventions. Body changes featured commonly in diet and physical activity interventions, restructuring the physical environment in alcohol interventions and providing feedback on behaviour in sexual health interventions and interventions targeting multiple behaviours. The BCTs shaping knowledge, comparison of outcomes, repetition and substitution, and the intervention function coercion were associated with lower cost-effectiveness, while the BCT reward and threat and intervention functions modelling and environmental restructuring, were associated with higher cost-effectiveness estimates.

Extensive evidence exists for the effectiveness of the most prevalent intervention functions and BCT clusters. In terms of education and shaping knowledge, educational materials have been shown to increase the uptake of cervical cancer screening [63] and to change attitudes towards excessive alcohol consumption [64]. However, providing knowledge and education alone is often not sufficient for enduring behaviour change. A phenomenon known as the knowledge-behaviour gap is commonly observed whereby what we believe we should do does not also tally with what we actually do in practice [65, 66]. For this reason, further support needs to be provided in the form of enablement (e.g. behavioural support and medications which are effective tools for helping smokers to stop [67]) or in the form of implementation intentions [68], which can involve action and goal planning one of the most commonly reported BCT clusters [69].

In our study, the wide variation in use of intervention functions across behavioral domains is unsurprising. For example, alcohol control policy has historically focused on reducing availability though licensing laws, minimum pricing for alcohol and age-of-sale restrictions [70]. In contrast, tobacco control has focused on a range of measures including educational and training approaches, in addition to coercive techniques such as increased taxation. In 1998 a national network of stop smoking services was set up in England with the aim of providing every smoker in the country who wanted help with stopping with access to evidence based behavioural support. This support includes the promotion of knowledge of the harms of smoking and training in relapse prevention, on top of a prescription for a smoking cessation medication, as recommended in clinical practice guidelines [71]. Persuasion has also been highly prevalent, most notably through the application of tobacco mass media campaigns [72, 73].

In our study, a relatively small number of BCTs were coded in the cost-effective interventions examined (an average of 10 out of a possible 93). This could be because only a small number of BCTs are effective or cost-effective, or because of insufficient intervention descriptions in published/available information [74]. Alternatively, it may reflect some intervention developers’ implicit theoretical assumptions regarding causes of behaviour and how it might be changed, or providers’ norms, historical bias and/or lack of training in intervention design [27, 75, 76]. Intervention design is also often governed and influenced by political and social priorities, and the goals of the funding source, which may impact on which BCTs are chosen [77, 78]. It is of particular interest that few interventions used BCTs based on operant learning (i.e the BCT clusters covert learning and reward and threat) [79]. This includes techniques which involve manipulation of environmental contingencies such as rewarding behaviour, using prompts and cues, agreeing on a behavioural contract and encouraging practice. Operant conditioning techniques have been applied successfully [80, 81] and are argued to underpin much of human behaviour [82, 83]. It is possible that interventions are inadvertently implementing such principles, with extinction and poor knowledge of schedules of reinforcement perhaps responsible for the failure to achieve maintainable behaviour change [83]. The fact that some BCTs were particularly common to specific health behaviour interventions is largely consistent with previous studies [29, 30] and can be systematically linked to theories of human behaviour [82, 84–86]. For example, the provision of social-support and goal-setting, which featured commonly in smoking cessation interventions, form part of Goal-Setting Theory [84], Social Learning Theory [85] and the Health Belief Model[86].

In this study, environmental restructuring, modelling of behaviour and threat and reward were associated with higher cost-effectiveness. The focus of interventions on removing or adding objects to the environment has been advocated by the popular book ‘Nudge’ [87]. Although it has proven efficacy [88], concerns have been raised that a reliance on its principles eschews the use of other efficacious BCW intervention functions [27]. Demonstration of behaviour and social comparison form part of several behaviour change theories including Social Comparison Theory and Social Learning Theory [85, 89], and have been associated previously with smoking cessation success [90], perception of alcohol-related negative consequences [91] and greater weight loss [92]. Threat and reward underpins the principles of Operant conditioning, which comprises some of the most underused BCTs’. Several factors influence the effectiveness of conditioning in practice, including the form of reward or punishment. Studies suggest that the optimal presentation of rewards should follow a ‘variable ratio schedule’ rather than ‘fixed ratio’ where a response is reinforced after an unpredictable number of responses [93].

In contrast, the function ‘coercion’ and the BCTs ‘shaping knowledge’, ‘comparison of outcomes’ and ‘repetition and substitution’, although present in cost-effective interventions, were associated with lower cost-effectiveness overall. ‘Coercion’ involves raising the financial cost of a behaviour whose incidence you wish to reduce. This might be through fiscal measures such as taxation or legislation. Intervention cost may increase with the inclusion of coercive methods as they place more emphasis on external influences which require manipulation than personal agency. However, there are instances where legislative measures can have a low per person cost compared with face-to-face interventions. There is substantial evidence for coercive measures in behaviour change. For example, raising the unit price of tobacco and alcohol products generates reductions in their use and healthcare costs [94, 95]. ‘Comparison of outcomes’ involves the use of persuasive arguments, summarising the pros and cons and comparative imagining of future outcomes, while ‘shaping knowledge’ covers instructions on how to perform a behaviour and use of antecedents and reattribution techniques. Finally, ‘repetition and substitution’ involves behavioural practice, substitution, habit formation or reversal and graded tasks. There are several explanations why these may incur higher cost, including the need for delivery by a trained professional and one-to-one support.

These findings have a number of implications. First, they may aid evidence-based practice and the application of BCIs in the public domain. Although part of the failure to implement interventions in the real world results from differences in choice of control conditions and resources, the ability to duplicate the components of the original intervention may also play a key role [19, 20], and this paper provides some of the key BCTs contained in cost-effective interventions. Secondly, studying the types of components of behavioural interventions in this manner may help enable scientific replication, by clearly specifying which components have been employed previously [33]. Replication is important both for ascertaining the generalisability of interventions and for increasing confidence in conclusions regarding their efficacy [96]. Thirdly, elucidating and summarising the components of cost-effective interventions may be a valuable resource to intervention designers, with guidelines recommending a full literature review of the components of efficacious interventions before development [97]. These findings may also help to encourage clearer reporting of intervention content and reveal gaps in the literature which need addressing [98]. For example, few studies used the BCT cluster coercion, reasons for this need to be identified and addressed. This is particularly important with the growing movement towards developing machine-readable papers based on highly specified ontologies to improve evidence synthesis [99]. Finally, this paper gives some indication as to the BCTs and intervention features which may be associated with greater cost-effectiveness. Future studies should aim to assess the contribution of these either in isolation or using factorial type designs.

To our knowledge, this is the first attempt to synthesise cost-effective BCIs in terms of their functions and ‘active ingredients’. However, this study also has several limitations. First, the BCT taxonomy coding approach was applied conservatively, in that a technique was coded as present only when there was unequivocal evidence from written materials that it was used. Because many intervention reports are poorly specified there was some uncertainty about inclusion of BCTs [29]. However, this approach did lead to greater specificity in identifying BCTs. Secondly, it is not possible to make a causal attribution of cost-effectiveness to specific BCTs. Although the regression analysis can be used to help discern these effects, caution should be taken because the BCIs typically contained many BCTs and the analyses were likely underpowered to detect small associations [100]. Thirdly, this paper did not compare effective with ineffective interventions or effective interventions which were cost-effective and not cost-effective, so we do not know how far these identified BCTs are unique to the interventions included in the current paper [88]. We also cannot make conclusions as to which BCTs may be ineffective or not cost-effective, only the degree to which they are cost-effective. It will be important to ascertain in future studies the features of interventions deemed ineffective and cost-ineffective in order to draw firmer conclusions. Nonetheless, this study provides, to our knowledge, the first indication of BCTs which are commonly applied in interventions deemed to be cost-effective in the United Kingdom (UK). Fourthly, this study focused on controlled studies, mainly RCTs, and many BCIs (e.g. tax increases) are not readily evaluated using this method. Fifthly, the process for identifying studies through using the NICE economic reports may have resulted in some interventions being missed and new studies are being published every week so the picture may change over time. Further studies are needed which assess the use of BCTs in other behavioural domains (e.g. cancer screening attendance and use of illicit drugs). Sixthly, for the behavioural domains other than smoking cessation only relatively small numbers were identified. This may reflect a tendency on the part of the research community not to conduct cost-effectiveness analyses for those behaviours. Several guidelines exist for the reporting of cost-effectiveness and researchers should be encouraged to follow these, with the quality of reporting of economic evaluations varying widely [101–103]. This has several implications, including the possibility that those interventions which fail to report such analyses are more likely to contain certain intervention functions and BCTs. Seventhly, this report is limited by the quality and time-frame of the economic analyses which provided the evidence-based for interventions included in this BCT analysis. Economic modelling itself is open to a number of limitations, such as uncertainty about temporal discounting, adjustment for quality of life and the use of disparate methodologies (e.g. assumptions) across reports [104]. This paper used the NICE threshold of cost effectiveness of £20,000-£30,000 per QALY. However, there is debate about the correct level of this threshold which should be used [105] and this varies enormously between countries [106] and even within countries. Eighthly, the adjusted model for the association with cost-effectiveness was derived using stepwise regression in order to prevent over-parameterization of the model. There are several criticisms of this procedure, including the production of biased regression coefficients and artificially narrow confidence intervals [107–110]. Alternative subjective methods such as hierarchical regression could not be used as we did not have any pre-specified theoretical hypotheses as to which associations may be present. Caution should also be taken when interpreting the results from the bivariate and adjusted regression models as they did not take into account sampling error. This can be achieved with meta-regression but unfortunately the sample sizes were too small for this technique [100]. Ninthly, a number of contextual features of interventions which may be associated with cost-effectiveness were not considered as they were beyond the scope of the current review. These include geographical location and more specific socio-demographic and cultural features of the samples included [111]. This will be an important avenue for future research. Finally, due to the small sample sizes for some of the analyses it is possible that we were underpowered to detect effects and associations. It will be important to update these findings as more literature becomes available.

In conclusion, this study reliably categorized and coded the BCTs used in cost-effective BCIs. These interventions heavily relied on education and enablement and most used relatively few BCTs. However, substantial variations were found in the content of interventions targeting the six health behaviour domains of interest, with alcohol interventions focusing less on education and enablement and more on restriction, and smoking interventions featuring most BCTs. There are a number of explanations for this, including the use of common sense models of human behaviour, poor reporting and variations in underlying etiology. Several intervention functions and BCTs were associated with higher cost-effectiveness: modelling, environmental restructuring and reward and threat. These findings will be of interest to intervention developers and policy makers attempting to implement BCIs in the real world.

## Supporting information

S1 Fig PRISMA Flow diagram. (DOCX)

S1 Material References for included studies. (DOCX)

S1 Table Most prevalent BCTs across the various health-behaviours. (DOCX)

S2 Table Broad categorisation of interventions. (DOCX)

S1 PRISMA Checklist (DOC)
