# Supplementary material for: What do cost-effective health behaviour-change interventions contain? A comparison of six domains
Source: PLoS One. 2019 Apr 17;14(4):e0213983. doi: 10.1371/journal.pone.0213983 (PMC6469762; doi:10.1371/journal.pone.0213983)
Supplement: S1 Fig — (DOCX) [file pone.0213983.s001.docx]

Retrieval of original studies where detail was lacking

(n=223)

Retrieval of relevant source documents from the 72 economic analyses

(n=115)

Excluded as cost-effectiveness estimates could not be derived

(n=7)

(n=7)

Interventions included in the guidance documents which considered cost-effectiveness

(n=79)

Identification of relevant NICE guidance documents

(n=19)

Papers reviewed and coded

(n=338)
