# Supplementary material for: What do cost-effective health behaviour-change interventions contain? A comparison of six domains
Source: PLoS One. 2019 Apr 17;14(4):e0213983. doi: 10.1371/journal.pone.0213983 (PMC6469762; doi:10.1371/journal.pone.0213983)
Supplement: S1 Material — (DOCX) [file pone.0213983.s002.docx]

Aalto, M., Saksanen, R., Laine, P., Forsstrom, R., Raikaa, M., Kiviluoto, M., Seppa, K., & Sillanaukee, P. 2000. Brief intervention for female heavy drinkers in routine general practice: a 3-year randomized, controlled study. *Alcohol Clin Exp.Res*, 24, 1680-1686.

Abelin, T., Buehler, A., Muller, P., Vesanen, K., & Imhof, P.R. 1989. Controlled trial of transdermal nicotine patch in tobacco withdrawal. *Lancet*, 1, 7-10.

Aittasalo, M., Miilunpalo, S., & Suni, J. 2004. The effectiveness of physical activity counseling in a work-site setting - A randomized, controlled trial. *Patient Education and Counseling*, 55, 193-202.

Albrecht, S., Payne, L., Stone, C.A., & Reynolds, M.D. 1998. A preliminary study of the use of peer support in smoking cessation programs for pregnant adolescents. *J.Am.Acad.Nurse Pract.*, 10, 119-125.

Altisent, R., Cordoba, R., Delgado, M.T., Pico, M.V., Melus, E., Aranguren, F., Alvira, U., Barbera, C., Moran, J., & Reixa, S. 1997. [Multicenter study on the efficacy of advice for the prevention of alcoholism in primary health care]. *Med Clin (Barc.)*, 109, 121-124.

Altman, D.G., Flora, J.A., Fortmann, S.P., & Farquhar, J.W. 1987. The cost-effectiveness of three smoking cessation programs. *Am J Public Health*, 77, 162-165.

An, L.C., Schillo, B.A., Kavanaugh, A.M., Lachter, R.B., Luxenberg, M.G., Wendling, A.H., & Joseph, A.M. 2006. Increased reach and effectiveness of a statewide tobacco quitline after the addition of access to free nicotine replacement therapy. *Tob.Control*, 15, 286-293.

Areechon, W. & Punnotok, J. 1988. Smoking cessation through the use of nicotine chewing gum: a double-blind trial in Thailand. *Clin.Ther.*, 10, 183-186.

Aveyard, P., Griffin, C., Lawrence, T., & Cheng, K.K. 2003. A controlled trial of an expert system and self-help manual intervention based on the stages of change versus standard self-help materials in smoking cessation. *Addiction*, 98, 345-354.

Avruch, S. & Cackley, A.P. 1995. Savings achieved by giving WIC benefits to women prenatally. *Public Health Rep.*, 110, 27-34.

Babor, T. F. a. G. M. e. Project on Identification and Management of Alcohol-Related Problems: Report on Phase Ii-a Randomised Clinical Trial of Brief Interventions in Primary Health Care. World Health Organisation, WHO/PSA/91.5. 1992.
Ref Type: Abstract

Bailey, L.B., Ofarrellray, B., Mahan, C.S., & Dimperio, D. 1983. Vitamin-B6, Iron and Folacin Status of Pregnant-Women. *Nutrition Research*, 3, 783-793.

Bains, N., Pickett, W., & Hoey, J. 1998. The use and impact of incentives in population-based smoking cessation programs: a review. *Am J Health Promot.*, 12, 307-320.

Barbeau, E.M., Li, Y., Calderon, P., Hartman, C., Quinn, M., Markkanen, P., Roelofs, C., Frazier, L., & Levenstein, C. 2006. Results of a union-based smoking cessation intervention for apprentice iron workers (United States). *Cancer Causes Control*, 17, 53-61.

Bauer, J.E., Carlin-Menter, S.M., Celestino, P.B., Hyland, A., & Cummings, K.M. 2006. Giving away free nicotine medications and a cigarette substitute (Better Quit) to promote calls to a quitline. *J.Public Health Manag.Pract.*, 12, 60-67.

Bauman, K.E., Bryan, E.S., Dent, C.W., & Koch, G.G. 1983. The influence of observing carbon monoxide level on cigarette smoking by public prenatal patients. *Am.J.Public Health*, 73, 1089-1091.

Baxter, A.P., Milner, P.C., Hawkins, S., Leaf, M., Simpson, C., Wilson, K.V., Owen, T., Higginbottom, G., Nicholl, J., & Cooper, N. 1997. The impact of heart health promotion on coronary heart disease lifestyle risk factors in schoolchildren: lessons learnt from a community-based project. *Public Health*, 111, 231-237.

Baxter, T., Milner, P., Wilson, K., Leaf, M., Nicholl, J., Freeman, J., & Cooper, N. 1997. A cost effective, community based heart health promotion project in England: prospective comparative study. *BMJ*, 315, 582-585.

Bentz, C.J., Bayley, K.B., Bonin, K.E., Fleming, L., Hollis, J.F., & McAfee, T. 2006. The feasibility of connecting physician offices to a state-level tobacco quit line. *Am J Prev Med*, 30, 31-37.

Betson, CL. A randomized controlled trial of smoking cessation in Government out-patient clinics in Hong Kong (Abstract OS 321). Proceedings of the 10th WorldConference on Tobacco orHealth; Aug 24-28; Beijing, China. 113. 1997.
Ref Type: Abstract

Betson CL. A randomized controlled trial on smoking cessation in government out-patient clinics in Hong Kong. Proceedings of the 10th World Conference on Tobacco or Health , 113. 24-8-1997.
Ref Type: Generic

Binnie, V.I., McHugh, S., Jenkins, W., Borland, W., & Macpherson, L.M. 2007. A randomised controlled trial of a smoking cessation intervention delivered by dental hygienists: a feasibility study. *BMC Oral Health*, 7, 5.

Blake, D. & Nied, A. 1997. The demand for alcohol in the United Kingdom. *Applied Economics*, 29, 1655-1672.

Blenkinsopp, A., Anderson, C., & Armstrong, M. 2003. Systematic review of the effectiveness of community pharmacy-based interventions to reduce risk behaviours and risk factors for coronary heart disease. *J.Public Health Med.*, 25, 144-153.

Blondal, T. 1989. Controlled trial of nicotine polacrilex gum with supportive measures. *Arch.Intern.Med.*, 149, 1818-1821.

Bolman, C., de, V.H., & van, B.G. 2002. Evaluation of a nurse-managed minimal-contact smoking cessation intervention for cardiac inpatients. *Health Educ.Res.*, 17, 99-116.

Bolu, O.O., Lindsey, C., Kamb, M.L., Kent, C., Zenilman, J., Douglas, J.M., Malotte, C.K., Rogers, J., & Peterman, T.A. 2004. Is HIV/sexually transmitted disease prevention counseling effective among vulnerable populations?: a subset analysis of data collected for a randomized, controlled trial evaluating counseling efficacy (Project RESPECT). *Sex Transm.Dis*, 31, 469-474.

Boyd, N.R., Sutton, C., Orleans, C.T., McClatchey, M.W., Bingler, R., Fleisher, L., Heller, D., Baum, S., Graves, C., & Ward, J.A. 1998. Quit Today! A targeted communications campaign to increase use of the cancer information service by African American smokers. *Prev Med*, 27, S50-S60.

Boyer, C.B., Barrett, D.C., Peterman, T.A., & Bolan, G. 1997. Sexually transmitted disease (STD) and HIV risk in heterosexual adults attending a public STD clinic: evaluation of a randomized controlled behavioral risk-reduction intervention trial. *AIDS*, 11, 359-367.

Brantmark, B., Ohlin, P., & Westling, H. 1973. Nicotine-containing chewing gum as an anti-smoking aid. *Psychopharmacologia.*, 31, 191-200.

British Thoracic Society. Comparison of four methods of smoking withdrawal in patients with smoking related diseases: Report by a subcommittee of the Research Committee of the British Thoracic Society. BMJ 286, 595-597. 1983.
Ref Type: Abstract

Buescher, P. 1991, *An evaluation of the impact of prenatal WIC participation on birth outcomes and Medicaid costs in North Carolina.*, North Carolina Department of Environment, Health and Natural Rescources, Report No. 55., NC.

Burling, T.A. Smoking during pregnancy: reduction via objective assessment and directive advice. Behavior Therapy 22, 31-40. 1991.
Ref Type: Abstract

Campbell, I.A., Hansford, M., & Prescott, R.J. 1986. Effect of a "stop smoking" booklet on smokers attending for chest radiography: a controlled study. *Thorax*, 41, 369-371.

Campbell, I.A., Lyons, E., & Prescott, R.J. 1987. Stopping smoking. Do nicotine chewing-gum and postal encouragement add to doctors' advice. *Practitioner*, 231, 114-117.

Campbell, N.C., Ritchie, L.D., Thain, J., Deans, H.G., Rawles, J.M., & Squair, J.L. 1998. Secondary prevention in coronary heart disease: a randomised trial of nurse led clinics in primary care. *Heart*, 80, 447-452.

Carpenter, C.S. & Eisenberg, D. 2009. Effects of Sunday Sales Restrictions on Overall and Day-Specific Alcohol Consumption: Evidence From Canada. *Journal of Studies on Alcohol and Drugs*, 70, 126-133.

Carr, A.B. & Ebbert, J.O. 2006. Interventions for tobacco cessation in the dental setting. *Cochrane Database Syst Rev* CD005084.

Chapman, S., Smith, W., Mowbray, G., Hugo, C., & Egger, G. 1993. Quit and win smoking cessation contests: how should effectiveness be evaluated? *Prev Med*, 22, 423-432.

Chisholm, D., Rehm, J., Van, O.M., & Monteiro, M. 2004. Reducing the global burden of hazardous alcohol use: a comparative cost-effectiveness analysis. *J Stud.Alcohol*, 65, 782-793.

Christen, A.G., McDonald, J.L., Jr., Olson, B.L., Drook, C.A., & Stookey, G.K. 1984. Efficacy of nicotine chewing gum in facilitating smoking cessation. *J.Am.Dent.Assoc.*, 108, 594-597.

Chyou, P.. Assessment of female participaton in an employee 20-week walking incentive program at Marshfiled clinic: a large multispeciality group practice. Clin Med Res 4, 256-265. 2006.
Ref Type: Abstract

Cinciripini, P.M., McClure, J.B., Wetter, D.W., Perry, J., Blalock, J.A., Cinciripini, L.G., Friedman, K.E., & Skaar, K. 2000. An evaluation of videotaped vignettes for smoking cessation and relapse prevention during pregnancy: the very important pregnant smokers (VIPS) program. *Tob.Control*, 9 Suppl 3, III61-III63.

Clavel, F., Benhamou, S., Company-Huertas, & Flamant, R. 1985. Helping people to stop smoking: randomised comparison of groups being treated with acupuncture and nicotine gum with control group. *Br.Med.J.(Clin.Res.Ed)*, 291, 1538-1539.

Cohen, S.J., Stookey, G.K., Katz, B.P., Drook, C.A., & Christen, A.G. 1989. Helping smokers quit: a randomized controlled trial with private practice dentists. *J.Am.Dent.Assoc.* , 118, 41-45.

Cohen, S.J., Stookey, G.K., Katz, B.P., Drook, C.A., & Smith, D.M. 1989. Encouraging primary care physicians to help smokers quit. A randomized, controlled trial. *Ann.Intern.Med.*, 110, 648-652.

Cope, A. The UK National Cycle Network: an assessment of the benefits of a sustainable transport infrastructure. World Transport Policy & Practice, 9(1), 6-17. 2003.
Ref Type: Abstract

Copeland, L., Robertson, R., & Elton, R. 2005. What happens when GPs proactively prescribe NRT patches in a disadvantaged community. *Scott.Med J*, 50, 64-68.

Cordoba, R., Delgado, M.T., Pico, V., Altisent, R., Fores, D., Monreal, A., Frisas, O., & Lopez, d., V 1998. Effectiveness of brief intervention on non-dependent alcohol drinkers (EBIAL): a Spanish multi-centre study. *Fam.Pract.*, 15 , 562-568.

Crawford, M.J., Patton, R., Touquet, R., Drummond, C., Byford, S., Barrett, B., Reece, B., Brown, A., & Henry, J.A. 2004. Screening and referral for brief intervention of alcohol-misusing patients in an emergency department: a pragmatic randomised controlled trial. *Lancet*, 364, 1334-1339.

Cuckle, H.S. & Van, V.H. 1984. The effectiveness of a postal smoking cessation 'kit'. *Community Med.*, 6, 210-215.

Cummings, K.M., Emont, S.L., Jaen, C., & Sciandra, R. 1988. Format and quitting instructions as factors influencing the impact of a self-administered quit smoking program. *Health Educ.Q.*, 15, 199-216.

Cummings, K.M., Kelly, J., Sciandra, R., Deloughry, T., & Francois, F. 1990. Impact of a community-wide stop smoking contest. *Am J Health Promot.*, 4, 429-434.

Curry, S.J., McBride, C., Grothaus, L.C., Louie, D., & Wagner, E.H. 1995. A randomized trial of self-help materials, personalized feedback, and telephone counseling with nonvolunteer smokers. *J Consult Clin Psychol.*, 63, 1005-1014.

Curry, S.J., McBride, C., Grothaus, L.C., Louie, D., & Wagner, E.H. 1995. A randomized trial of self-help materials, personalized feedback, and telephone counseling with nonvolunteer smokers. *J.Consult Clin.Psychol.*, 63, 1005-1014.

Curry, S.J., Ludman, E.J., Graham, E., Stout, J., Grothaus, L., & Lozano, P. 2003. Pediatric-based smoking cessation intervention for low-income women: a randomized trial. *Arch Pediatr.Adolesc.Med*, 157, 295-302.

Curry, S.J., Ludman, E.J., Grothaus, L.C., Donovan, D., & Kim, E. 2003. A randomized trial of a brief primary-care-based intervention for reducing at-risk drinking practices. *Health Psychol.*, 22, 156-165.

Daughton, D., Susman, J., Sitorius, M., Belenky, S., Millatmal, T., Nowak, R., Patil, K., & Rennard, S.I. 1998. Transdermal nicotine therapy and primary care. Importance of counseling, demographic, and participant selection factors on 1-year quit rates. The Nebraska Primary Practice Smoking Cessation Trial Group. *Arch.Fam.Med.*, 7, 425-430.

Daughton, D.M., Heatley, S.A., Prendergast, J.J., Causey, D., Knowles, M., Rolf, C.N., Cheney, R.A., Hatlelid, K., Thompson, A.B., & Rennard, S.I. 1991. Effect of transdermal nicotine delivery as an adjunct to low-intervention smoking cessation therapy. A randomized, placebo-controlled, double-blind study. *Arch.Intern.Med.*, 151, 749-752.

Davidson, M., Epstein, M., Burt, R., Schaefer, C., Whitworth, G., & McDonald, A. 1998. Efficacy and safety of an over-the-counter transdermal nicotine patch as an aid for smoking cessation. *Arch.Fam.Med.*, 7, 569-574.

Davies, B.L., Matte-Lewis, L., O'Connor, A.M., Dulberg, C.S., & Drake, E.R. 1992. Evaluation of the "Time to Quit" self-help smoking cessation program. *Can.J.Public Health*, 83, 19-23.

Davis, A.L., Faust, R., & Ordentlich, M. 1984. Self-help smoking cessation and maintenance programs: a comparative study with 12-month follow-up by the American Lung Association. *Am.J.Public Health*, 74, 1212-1217.

Davis, S.W., Cummings, K.M., Rimer, B.K., Sciandra, R., & Stone, J.C. 1992. The impact of tailored self-help smoking cessation guides on young mothers. *Health Educ.Q.*, 19, 495-504.

DeBusk, R.F., Miller, N.H., Superko, H.R., Dennis, C.A., Thomas, R.J., Lew, H.T., Berger, W.E., III, Heller, R.S., Rompf, J., Gee, D., Kraemer, H.C., Bandura, A., Ghandour, G., Clark, M., Shah, R.V., Fisher, L., & Taylor, C.B. 1994. A case-management system for coronary risk factor modification after acute myocardial infarction. *Ann.Intern.Med*, 120, 721-729.

Demers, R.Y., Neale, A.V., Adams, R., Trembath, C., & Herman, S.C. 1990. The impact of physicians' brief smoking cessation counseling: a MIRNET study. *J.Fam.Pract.*, 31, 625-629.

Diez, J. Brief intervention in Cantabria (Spain) in alcohol related problems. Adicciones 22, 156-165. 202.

Dijkstra, A., De, V.H., & Roijackers, J. 1998. Long-term effectiveness of computer-generated tailored feedback in smoking cessation. *Health Educ.Res.*, 13, 207-214.

Dijkstra, A., De, V.H., Roijackers, J., & van, B.G. 1998. Tailoring information to enhance quitting in smokers with low motivation to quit: three basic efficacy questions. *Health Psychol.*, 17, 513-519.

Dijkstra, A., De, V.H., & Roijackers, J. 1999. Targeting smokers with low readiness to change with tailored and nontailored self-help materials. *Prev.Med.*, 28, 203-211.

Donatelle, R.J., Prows, S.L., Champeau, D., & Hudson, D. 2000. Randomised controlled trial using social support and financial incentives for high risk pregnant smokers: significant other supporter (SOS) program. *Tob.Control*, 9 Suppl 3, III67-III69.

Dornelas, E.A., Magnavita, J., Beazoglou, T., Fischer, E.H., Oncken, C., Lando, H., Greene, J., Barbagallo, J., Stepnowski, R., & Gregonis, E. 2006. Efficacy and cost-effectiveness of a clinic-based counseling intervention tested in an ethnically diverse sample of pregnant smokers. *Patient.Educ.Couns.*, 64, 342-349.

Dunkley, J. 1997. Training midwives to help pregnant women stop smoking. *Nurs.Times*, 93, 64-66.

Ebbert, J.O., Carr, A.B., Patten, C.A., Morris, R.A., & Schroeder, D.R. 2007. Tobacco use quitline enrollment through dental practices: a pilot study. *J Am Dent Assoc*, 138, 595-601.

Eisen, M., Zellman, G.L., Massett, H.A., & Murray, D.M. 2002. Evaluating the Lions-Quest "Skills for Adolescence" drug education program: first-year behavior outcomes. *Addict.Behav.*, 27, 619-632.

Eisen, M., Zellman, G.L., & Murray, D.M. 2003. Evaluating the Lions-Quest "Skills for Adolescence" drug education program. Second-year behavior outcomes. *Addict.Behav.*, 28, 883-897.

Elder, J.P., McGraw, S.A., Rodrigues, A., Lasater, T.M., Ferreira, A., Kendall, L., Peterson, G., & Carleton, R.A. 1987. Evaluation of two community-wide smoking cessation contests. *Prev Med*, 16, 221-234.

Elder, J.P., Campbell, N.R., Mielchen, S.D., Hovell, M.F., & Litrownik, A.J. 1991. Implementation and evaluation of a community-sponsored smoking cessation contest. *Am J Health Promot.*, 5, 200-207.

Elley, C.R., Kerse, N., Arroll, B., & Robinson, E. 2003. Effectiveness of counselling patients on physical activity in general practice: cluster randomised controlled trial. *BMJ*, 326, 793.

Ershoff, D.H., Quinn, V.P., & Mullen, P.D. 1995. Relapse prevention among women who stop smoking early in pregnancy: a randomized clinical trial of a self-help intervention. *Am.J.Prev.Med.*, 11, 178-184.

Ershoff, D.H., Quinn, V.P., Boyd, N.R., Stern, J., Gregory, M., & Wirtschafter, D. 1999. The Kaiser Permanente prenatal smoking-cessation trial: when more isn't better, what is enough? *Am.J.Prev.Med.*, 17, 161-168.

Fagerstrom, K.O. 1983. Tolerance, withdrawal and dependence on tobacco and smoking termination. *Applied Psychology*, 32, 29-52.

Fagerstrom, K.O. 1984. Effects of nicotine chewing gum and follow-up appointments in physician-based smoking cessation. *Prev.Med.*, 13, 517-527.

Farquhar, J.W., Fortmann, S.P., Flora, J.A., Taylor, C.B., Haskell, W.L., Williams, P.T., Maccoby, N., & Wood, P.D. 1990. Effects of communitywide education on cardiovascular disease risk factors. The Stanford Five-City Project. *JAMA*, 264, 359-365.

Fee, W.M. & Stewart, M.J. 1982. A controlled trial of nicotine chewing gum in a smoking withdrawal clinic.  *Practitioner*, 226, 148-151.

Fernandez San Martin, M.I. Effectiveness of brief medical counseling to reduce drinkers' alcohol consumption. Atencion Primaria 19, 127-132. 1997.
Ref Type: Abstract

Finkelstein, E.A., Troped, P.J., Will, J.C., & Palombo, R. 2002. Cost-effectiveness of a cardiovascular disease risk reduction program aimed at financially vulnerable women: the Massachusetts WISEWOMAN project. *J Womens Health Gend.Based.Med*, 11, 519-526.

Fiscella, K. & Franks, P. 1996. Cost-effectiveness of the transdermal nicotine patch as an adjunct to physicians' smoking cessation counseling. *JAMA*, 275, 1247-1251.

Fleming, M., Brown, R., & Brown, D. 2004. The efficacy of a brief alcohol intervention combined with %CDT feedback in patients being treated for type 2 diabetes and/or hypertension. *J Stud.Alcohol*, 65, 631-637.

Fleming, M.F., Manwell, L.B., Barry, K.L., Adams, W., & Stauffacher, E.A. 1999. Brief physician advice for alcohol problems in older adults: a randomized community-based trial. *J Fam.Pract.*, 48, 378-384.

Fleming, M.F., Manwell, L.B., Barry, K.L., Adams, W., & Stauffacher, E.A. 1999. Brief physician advice for alcohol problems in older adults: a randomized community-based trial. *J Fam.Pract*, 48, 378-384.

Fortmann, S.P., Williams, P.T., Hulley, S.B., Haskell, W.L., & Farquhar, J.W. 1981. Effect of health education on dietary behavior: the Stanford Three Community Study. *Am.J Clin Nutr.*, 34, 2030-2038.

Fortmann, S.P. & Killen, J.D. 1995. Nicotine gum and self-help behavioral treatment for smoking relapse prevention: results from a trial using population-based recruitment. *J Consult Clin Psychol.*, 63, 460-468.

Fortmann, S.P. & Killen, J.D. 1995. Nicotine gum and self-help behavioral treatment for smoking relapse prevention: results from a trial using population-based recruitment. *J.Consult Clin.Psychol.*, 63, 460-468.

Gallet, C.A. 2007. The demand for alcohol: a meta-analysis of elasticities. *Australian Journal of Agricultural and Resource Economics*, 51, 121-135.

Gentilello, M. R. Alcohol interventions in a trauma center as a means of reducing the risk of injury recurrence. Annals of Surgery 230, 473-483. 1999.
Ref Type: Abstract

Gielen, A.C., Windsor, R., Faden, R.R., O'Campo, P., Repke, J., & Davis, M. 1997. Evaluation of a smoking cessation intervention for pregnant women in an urban prenatal clinic. *Health Educ.Res.*, 12, 247-254.

Gilbert, J.R., Wilson, D.M., Best, J.A., Taylor, D.W., Lindsay, E.A., Singer, J., & Willms, D.G. 1989. Smoking cessation in primary care. A randomized controlled trial of nicotine-bearing chewing gum. *J.Fam.Pract.*, 28, 49-55.

Glasgow, R.E., Klesges, R.C., Mizes, J.S., & Pechacek, T.F. 1985. Quitting smoking: strategies used and variables associated with success in a stop-smoking contest. *J Consult Clin Psychol.*, 53, 905-912.

Glavas, D. & Rumboldt, Z. 2003. [Smoking cessation using the transdermal nicotine system]. *Lijec.Vjesn.*, 125, 8-12.

Glavas, D., Rumboldt, M., & Rumboldt, Z. 2003. Smoking cessation with nicotine replacement therapy among health care workers: randomized double-blind study. *Croat.Med.J.*, 44, 219-224.

Golden, M.R., Whittington, W.L., Handsfield, H.H., Hughes, J.P., Stamm, W.E., Hogben, M., Clark, A., Malinski, C., Helmers, J.R., Thomas, K.K., & Holmes, K.K. 2005. Effect of expedited treatment of sex partners on recurrent or persistent gonorrhea or chlamydial infection. *N.Engl.J Med*, 352, 676-685.

Golden, M.R., Gift, T.L., Brewer, D.D., Fleming, M., Hogben, M., St Lawrence, J.S., Thiede, H., & Handsfield, H.H. 2006. Peer referral for HIV case-finding among men who have sex with men. *AIDS*, 20, 1961-1968.

Goldfield, G.S., Epstein, L.H., Kilanowski, C.K., Paluch, R.A., & Kogut-Bossler, B. 2001. Cost-effectiveness of group and mixed family-based treatment for childhood obesity. *Int.J Obes.Relat Metab Disord.*, 25, 1843-1849.

Gordon, J.S., Andrews, J.A., Crews, K.M., Payne, T.J., Severson, H.H., & Lichtenstein, E. 2010. Do faxed quitline referrals add value to dental office-based tobacco-use cessation interventions? *J Am Dent Assoc*, 141, 1000-1007.

Gordon, J.S., Andrews, J.A., Albert, D.A., Crews, K.M., Payne, T.J., & Severson, H.H. 2010. Tobacco cessation via public dental clinics: results of a randomized trial. *Am J Public Health*, 100, 1307-1312.

Gray, C. M. Addressing male obesity: an evaluation of a group-based weight management intervention for Scottish men. Journal of Men's Health 6, 70-81. 2009.
Ref Type: Abstract

Gritz, E.R., Berman, B.A., Bastani, R., & Wu, M. 1992. A randomized trial of a self-help smoking cessation intervention in a nonvolunteer female population: testing the limits of the public health model. *Health Psychol.*, 11, 280-289.

Gruenewald, P.J., Ponicki, W.R., & Holder, H.D. 1993. The Relationship of Outlet Densities to Alcohol-Consumption - A Time-Series Cross-Sectional Analysis. *Alcoholism-Clinical and Experimental Research*, 17, 38-47.

Gruenewald, P.J., Ponicki, W.R., Holder, H.D., & Romelsjo, A. 2006. Alcohol prices, beverage quality, and the demand for alcohol: Quality substitutions and price elasticities. *Alcoholism-Clinical and Experimental Research*, 30, 96-105.

Hajek, P., Taylor, T.Z., & Mills, P. 2002. Brief intervention during hospital admission to help patients to give up smoking after myocardial infarction and bypass surgery: randomised controlled trial. *BMJ*, 324, 87-89.

Hall, S., Bishop, A.J., & Marteau, T.M. 2003. Increasing readiness to stop smoking in women undergoing cervical screening: evaluation of two leaflets. *Nicotine Tob Res*, 5, 821-826.

Hall, S., Reid, E., Ukoumunne, O.C., Weinman, J., & Marteau, T.M. 2007. Brief smoking cessation advice from practice nurses during routine cervical smear tests appointments: a cluster randomised controlled trial assessing feasibility, acceptability and potential effectiveness. *Br J Cancer*, 96, 1057-1061.

Hall, S.M. & Killen, J.D. 1985. Psychological and pharmacological approaches to smoking relapse prevention. *NIDA Res.Monogr*, 53, 131-143.

Hall, S.M., Tunstall, C.D., Ginsberg, D., Benowitz, N.L., & Jones, R.T. 1987. Nicotine gum and behavioral treatment: a placebo controlled trial. *J.Consult Clin.Psychol.*, 55, 603-605.

Hanioka, T., Ojima, M., Tanaka, H., Naito, M., Hamajima, N., & Matsuse, R. 2010. Intensive smoking-cessation intervention in the dental setting. *J Dent Res*, 89, 66-70.

Harackiewicz, J.M., Blair, L.W., Sansone, C., Epstein, J.A., & Stuchell, R.N. 1988. Nicotine gum and self-help manuals in smoking cessation: an evaluation in a medical context. *Addict.Behav.*, 13, 319-330.

Harding, R., Bensley, J., & Corrigan, N. 2004. Targeting smoking cessation to high prevalence communities: outcomes from a pilot intervention for gay men. *BMC Public Health*, 4, 43.

Harland, J., White, M., Drinkwater, C., Chinn, D., Farr, L., & Howel, D. 1999. The Newcastle exercise project: a randomised controlled trial of methods, to promote physical activity in primary care. *British Medical Journal*, 319, 828-832B.

Haug, K., Fugelli, P., Aaro, L.E., & Foss, O.P. 1994. Is smoking intervention in general practice more successful among pregnant than non-pregnant women? *Fam.Pract.*, 11, 111-116.

Hays, J.T., Croghan, I.T., Schroeder, D.R., Offord, K.P., Hurt, R.D., Wolter, T.D., Nides, M.A., & Davidson, M. 1999. Over-the-counter nicotine patch therapy for smoking cessation: results from randomized, double-blind, placebo-controlled, and open label trials. *Am.J.Public Health*, 89, 1701-1707.

Heather, N., Campion, P.D., Neville, R.G., & Maccabe, D. 1987. Evaluation of a controlled drinking minimal intervention for problem drinkers in general practice (the DRAMS scheme). *J R.Coll.Gen Pract*, 37, 358-363.

Hegaard, H.K., Kjaergaard, H., Moller, L.F., Wachmann, H., & Ottesen, B. 2003. Multimodal intervention raises smoking cessation rate during pregnancy. *Acta Obstet.Gynecol.Scand.*, 82, 813-819.

Hennrikus, D.J., Jeffery, R.W., Lando, H.A., Murray, D.M., Brelje, K., Davidann, B., Baxter, J.S., Thai, D., Vessey, J., & Liu, J. 2002. The SUCCESS project: the effect of program format and incentives on participation and cessation in worksite smoking cessation programs. *Am.J.Public Health*, 92, 274-279.

Hennrikus, D.J., Lando, H.A., McCarty, M.C., Klevan, D., Holtan, N., Huebsch, J.A., Jestus, S., Pentel, P.R., Pine, D., Sullivan, S., Swenson, K., & Vessey, J. 2005. The TEAM project: the effectiveness of smoking cessation intervention with hospital patients. *Prev.Med.*, 40, 249-258.

Higashi, A. Efficacy of smoking cessation instruction for general smokers at an annual physical examination. Nippon Koshu EiseiZasshi 42, 313-321. 1995.
Ref Type: Generic

Hillsdon, M., Thorogood, M., White, I., & Foster, C. 2002. Advising people to take more exercise is ineffective: a randomized controlled trial of physical activity promotion in primary care. *International Journal of Epidemiology*, 31, 808-815.

Hjalmarson, A.I. 1984. Effect of nicotine chewing gum in smoking cessation. A randomized, placebo-controlled, double-blind study. *JAMA*, 252, 2835-2838.

Hjalmarson, A.I., Hahn, L., & Svanberg, B. 1991. Stopping smoking in pregnancy: effect of a self-help manual in controlled trial. *Br.J.Obstet.Gynaecol.*, 98, 260-264.

Hoadley, J.F., Fuchs, B.C., & Holder, H.D. 1984. The Effect of Alcohol Beverage Restrictions on Consumption - A 25-Year Longitudinal Analysis. *American Journal of Drug and Alcohol Abuse*, 10, 375-401.

Hollis, J.F., Lichtenstein, E., Vogt, T.M., Stevens, V.J., & Biglan, A. 1993. Nurse-assisted counseling for smokers in primary care. *Ann.Intern.Med.*, 118, 521-525.

Huas, D. Efficacy of a brief intervention for problematic alcohol users[Efficacite a un an d'une intervention breve aupres des consommateurs d'alcool a problemes. Rev Pract Med Gen 16, 1343-1348. 2002.
Ref Type: Abstract

Huber, D. 1988. Combined and separate treatment effects of nicotine chewing gum and self-control method. *Pharmacopsychiatry*, 21, 461-462.

Hudmon, K.S., Gritz, E.R., Clayton, S., & Nisenbaum, R. 1999. Eating orientation, postcessation weight gain, and continued abstinence among female smokers receiving an unsolicited smoking cessation intervention. *Health Psychol.*, 18 , 29-36.

Hughes, J.R., Gust, S.W., Keenan, R.M., Fenwick, J.W., & Healey, M.L. 1989. Nicotine vs placebo gum in general medical practice. *JAMA*, 261, 1300-1305.

Hughes, J.R., Gulliver, S.B., Amori, G., Mireault, G.C., & Fenwick, J.F. 1989. Effect of instructions and nicotine on smoking cessation, withdrawal symptoms and self-administration of nicotine gum. *Psychopharmacology (Berl)*, 99, 486-491.

Hughes, J.R., Gust, S.W., Keenan, R.M., & Fenwick, J.W. 1990. Effect of dose on nicotine's reinforcing, withdrawal-suppression and self-reported effects. *J.Pharmacol.Exp.Ther.* , 252, 1175-1183.

Humerfelt, S., Eide, G.E., Kvale, G., Aaro, L.E., & Gulsvik, A. 1998. Effectiveness of postal smoking cessation advice: a randomized controlled trial in young men with reduced FEV1 and asbestos exposure. *Eur.Respir.J.*, 11, 284-290.

James, N.J., Gillies, P.A., & Bignell, C.J. 1998. Evaluation of a randomized controlled trial of HIV and sexually transmitted disease prevention in a genitourinary medicine clinic setting. *AIDS*, 12, 1235-1242.

Jamrozik, K., Vessey, M., Fowler, G., Wald, N., Parker, G., & Van, V.H. 1984. Controlled trial of three different antismoking interventions in general practice. *Br.Med.J.(Clin.Res.Ed)*, 288, 1499-1503.

Jamrozik, K., Fowler, G., Vessey, M., & Wald, N. 1984. Placebo controlled trial of nicotine chewing gum in general practice. *Br.Med.J.(Clin.Res.Ed)*, 289, 794-797.

Janz, N.K., Becker, M.H., Kirscht, J.P., Eraker, S.A., Billi, J.E., & Woolliscroft, J.O. 1987. Evaluation of a minimal-contact smoking cessation intervention in an outpatient setting. *Am.J.Public Health*, 77, 805-809.

Jarvik, M.E. & Schneider, N.G. 1984. Degree of addiction and effectiveness of nicotine gum therapy for smoking. *Am.J.Psychiatry*, 141, 790-791.

Jarvis, M.J., Raw, M., Russell, M.A., & Feyerabend, C. 1982. Randomised controlled trial of nicotine chewing-gum. *Br Med J (Clin Res Ed)*, 285, 537-540.

Javitz, H.S., Swan, G.E., Zbikowski, S.M., Curry, S.J., McAfee, T.A., Decker, D., Patterson, R., & Jack, L.M. 2004. Return on investment of different combinations of bupropion SR dose and behavioral treatment for smoking cessation in a health care setting: an employer's perspective. *Value.Health*, 7, 535-543.

Kalichman, S.C., Cain, D., Weinhardt, L., Benotsch, E., Presser, K., Zweben, A., Bjodstrup, B., & Swain, G.R. 2005. Experimental components analysis of brief theory-based HIV/AIDS risk-reduction counseling for sexually transmitted infection patients. *Health Psychol.*, 24, 198-208.

Kamb, M.L., Fishbein, M., Douglas, J.M., Jr., Rhodes, F., Rogers, J., Bolan, G., Zenilman, J., Hoxworth, T., Malotte, C.K., Iatesta, M., Kent, C., Lentz, A., Graziano, S., Byers, R.H., & Peterman, T.A. 1998. Efficacy of risk-reduction counseling to prevent human immunodeficiency virus and sexually transmitted diseases: a randomized controlled trial. Project RESPECT Study Group. *JAMA*, 280, 1161-1167.

Kaner, E.F., Beyer, F., Dickinson, H.O., Pienaar, E., Campbell, F., Schlesinger, C., Heather, N., Saunders, J., & Burnand, B. 2007. Effectiveness of brief alcohol interventions in primary care populations. *Cochrane Database Syst.Rev.* CD004148.

Kennedy, E.T., Gershoff, S., Reed, R., & Austin, J.E. 1982. Evaluation of the effect of WIC supplemental feeding on birth weight. *J.Am.Diet.Assoc.*, 80, 220-227.

Kennedy, E.T. & Kotelchuck, M. 1984. The effect of WIC supplemental feeding on birth weight: a case-control analysis. *Am.J.Clin.Nutr.*, 40, 579-585.

Killen, J.D., Fortmann, S.P., Newman, B., & Varady, A. 1990. Evaluation of a treatment approach combining nicotine gum with self-guided behavioral treatments for smoking relapse prevention. *J.Consult Clin.Psychol.*, 58, 85-92.

Killen, J.D., Fortmann, S.P., Davis, L., & Varady, A. 1997. Nicotine patch and self-help video for cigarette smoking cessation. *J.Consult Clin.Psychol.*, 65, 663-672.

Killen, J.D., Maccoby, N., & Taylor, C.B. 1984. Nicotine gum and self-regulation training in smoking relapse prevention. *Behavior Therapy*, 15, 234-248.

King, A.C., Flora, J.A., Fortmann, S.P., & Taylor, C.B. 1987. Smokers' challenge: immediate and long-term findings of a community smoking cessation contest. *Am J Public Health* , 77, 1340-1341.

Korhonen, H.J., Niemensivu, H., Piha, T., Koskela, K., Wiio, J., Johnson, C.A., & Puska, P. 1992. National TV smoking cessation program and contest in Finland. *Prev Med*, 21, 74-87.

Korhonen, H.J., Puska, P., Lipand, A., & Kasmel, A. 1993. Combining mass media and contest in smoking cessation. An experience from a series of national activities in Finland. *Hygie*, 12, 14-18.

Kotelchuck, M., Schwartz, J.B., Anderka, M.T., & Finison, K.S. 1984. WIC participation and pregnancy outcomes: Massachusetts Statewide Evaluation Project. *Am.J.Public Health*, 74, 1086-1092.

Kottke, T.E., Brekke, M.L., Solberg, L.I., & Hughes, J.R. 1989. A randomized trial to increase smoking intervention by physicians. Doctors Helping Smokers, Round I. *JAMA*, 261, 2101-2106.

Kunz, F.M., Jr., French, M.T., & Bazargan-Hejazi, S. 2004. Cost-effectiveness analysis of a brief intervention delivered to problem drinkers presenting at an inner-city hospital emergency department. *J Stud.Alcohol*, 65, 363-370.

Lamb, S.E., Bartlett, H.P., Ashley, A., & Bird, W. 2002. Can lay-led walking programmes increase physical activity in middle aged adults? A randomised controlled trial. *Journal of Epidemiology and Community Health*, 56, 246-252.

Lancaster, T. & Stead, L.F. 2005. Self-help interventions for smoking cessation. *Cochrane Database Syst.Rev.* CD001118.

Lando, H.A. Promoting tobacco abstinence among older adolescents in dental clinics. Journal of Smoking Cessation 2, 23-30. 2007.
Ref Type: Abstract

Lando, H. Recycling of chronic smokers to sustained abstinence. Ninth World Conference on Tobacco and Health, Paris, France . 1994.
Ref Type: Abstract

Lando, H.A., Kalb, E.A., & McGovern, P.G. 1988. Behavioral self-help materials as an adjunct to nicotine gum. *Addict.Behav.*, 13, 181-184.

Lando, H.A., McGovern, P.G., Barrios, F.X., & Etringer, B.D. 1990. Comparative evaluation of American Cancer Society and American Lung Association smoking cessation clinics. *Am J Public Health*, 80, 554-559.

Lando, H.A., McGovern, P.G., Barrios, F.X., & Etringer, B.D. 1990. Comparative evaluation of American Cancer Society and American Lung Association smoking cessation clinics. *Am.J.Public Health*, 80, 554-559.

Lando, H.A., Pirie, P.L., McGovern, P.G., Pechacek, T.F., Swim, J., & Loken, B. 1991. A comparison of self-help approaches to smoking cessation. *Addict.Behav.*, 16, 183-193.

Lando, H.A., Hellerstedt, W.L., Pirie, P.L., & McGovern, P.G. 1992. Brief supportive telephone outreach as a recruitment and intervention strategy for smoking cessation. *Am J Public Health*, 82, 41-46.

Lando, H.A., Rolnick, S., Klevan, D., Roski, J., Cherney, L., & Lauger, G. 1997. Telephone support as an adjunct to transdermal nicotine in smoking cessation. *Am J Public Health*, 87, 1670-1674.

Langham, S., Thorogood, M., Normand, C., Muir, J., Jones, L., & Fowler, G. 1996. Costs and cost effectiveness of health checks conducted by nurses in primary care: the Oxcheck study. *BMJ*, 312, 1265-1268.

Lazev, A., Vidrine, D., Arduino, R., & Gritz, E. 2004. Increasing access to smoking cessation treatment in a low-income, HIV-positive population: the feasibility of using cellular telephones. *Nicotine Tob Res*, 6, 281-286.

Ledwith, F. 1984. Immediate and delayed effects of postal advice on stopping smoking. *Health Bull.(Edinb.)*, 42, 332-344.

Lee, M.E., Lichtenstein, E., Andrews, J.A., Glasgow, R.E., & Hampson, S.E. 1999. Radon-smoking synergy: A population-based behavioral risk reduction approach. *Prev.Med.*, 29, 222-227.

Lefebvre, R.C., Cobb, G.D., Goreczny, A.J., & Carleton, R.A. 1990. Efficacy of an incentive-based community smoking cessation program. *Addict.Behav*, 15 , 403-411.

Leinweber, C.E., Macdonald, J.M., & Campbell, H.S. 1994. Community smoking cessation contests: an effective public health strategy. *Can.J Public Health*, 85, 95-98.

Lennox, A.S., Osman, L.M., Reiter, E., Robertson, R., Friend, J., McCann, I., Skatun, D., & Donnan, P.T. 2001. Cost effectiveness of computer tailored and non-tailored smoking cessation letters in general practice: randomised controlled trial. *BMJ*, 322, 1396.

Leppanen, K. lauantaisulkemiskokoeilun vaikutuksista alkoholijuomien myyntiin (Effects of national Saturday closing experiment on alcohol sales). Alkoholipolitiikka 44, 20-21. 1979.
Ref Type: Abstract

Leslie, E. University campus physical activity promotion program. Health Promotion Journal of Australia 10, 51-54. 2000.
Ref Type: Abstract

Levy, D.T. & Miller, T.R. 1995. A cost-benefit analysis of enforcement efforts to reduce serving intoxicated patrons. *J Stud.Alcohol*, 56, 240-247.

Lichtenstein, E., Glasgow, R.E., Lando, H.A., Ossip-Klein, D.J., & Boles, S.M. 1996. Telephone counseling for smoking cessation: rationales and meta-analytic review of evidence. *Health Educ.Res*, 11, 243-257.

Lichtenstein, E., Andrews, J.A., Lee, M.E., Glasgow, R.E., & Hampson, S.E. 2000. Using radon risk to motivate smoking reduction: evaluation of written materials and brief telephone counselling. *Tob.Control*, 9, 320-326.

Lindgren, P., Fahlstadius, P., Hellenius, M.L., Jonsson, B., & de, F.U. 2003. Cost-effectiveness of primary prevention of coronary heart disease through risk factor intervention in 60-year-old men from the county of Stockholm--a stochastic model of exercise and dietary advice. *Prev Med*, 36, 403-409.

Lindholm, L., Rosen, M., Weinehall, L., & Asplund, K. 1996. Cost effectiveness and equity of a community based cardiovascular disease prevention programme in Norsjo, Sweden. *J Epidemiol.Community Health*, 50, 190-195.

Lock, C.A., Kaner, E., Heather, N., Doughty, J., Crawshaw, A., McNamee, P., Purdy, S., & Pearson, P. 2006. Effectiveness of nurse-led brief alcohol intervention: a cluster randomized controlled trial. *J Adv.Nurs*, 54, 426-439.

Lovelady, C.A., Williams, J.P., Garner, K.E., Moreno, K.L., Taylor, M.L., & Leklem, J.E. 2001. Effect of energy restriction and exercise on vitamin B-6 status of women during lactation.  *Medicine and Science in Sports and Exercise*, 33, 512-518.

Lovelady, C.A., Stephenson, K.G., Kuppler, K.M., & Williams, J.P. 2006. The effects of dieting on food and nutrient intake of lactating women. *Journal of the American Dietetic Association*, 106, 908-912.

Low, N., McCarthy, A., Roberts, T.E., Huengsberg, M., Sanford, E., Sterne, J.A., Macleod, J., Salisbury, C., Pye, K., Holloway, A., Morcom, A., Patel, R., Robinson, S.M., Horner, P., Barton, P.M., & Egger, M. 2006. Partner notification of chlamydia infection in primary care: randomised controlled trial and analysis of resource use. *BMJ*, 332, 14-19.

Lowe, J.B., Windsor, R., Balanda, K.P., & Woodby, L. 1997. Smoking relapse prevention methods for pregnant women: a formative evaluation. *Am.J.Health Promot.*, 11, 244-246.

Lowey, H., Tocque, K., Bellis, M.A., & Fullard, B. 2003. Smoking cessation services are reducing inequalities. *J Epidemiol Community Health*, 57, 579-580.

Lumley, J., Chamberlain, C., Dowswell, T., Oliver, S., Oakley, L., & Watson, L. 2009. Interventions for promoting smoking cessation during pregnancy. *Cochrane Database Syst.Rev.* CD001055.

Maguire, T.A., McElnay, J.C., & Drummond, A. 2001. A randomized controlled trial of a smoking cessation intervention based in community pharmacies. *Addiction*, 96, 325-331.

Maher, J.E., Peterman, T.A., Osewe, P.L., Odusanya, S., & Scerba, J.R. 2003. Evaluation of a community-based organization's intervention to reduce the incidence of sexually transmitted diseases: a randomized, controlled trial. *South Med J*, 96, 248-253.

Maisto, S.A., Conigliaro, J., McNeil, M., Kraemer, K., Conigliaro, R.L., & Kelley, M.E. 2001. Effects of two types of brief intervention and readiness to change on alcohol use in hazardous drinkers. *J Stud.Alcohol*, 62, 605-614.

Malchodi, C.S., Oncken, C., Dornelas, E.A., Caramanica, L., Gregonis, E., & Curry, S.L. 2003. The effects of peer counseling on smoking cessation and reduction. *Obstet.Gynecol.*, 101, 504-510.

Malcolm, R.E., Sillett, R.W., Turner, J.A., & Ball, K.P. 1980. The use of nicotine chewing gum as an aid to stopping smoking. *Psychopharmacology (Berl)*, 70, 295-296.

Mansdotter, A.M., Rydberg, M.K., Wallin, E., Lindholm, L.A., & Andreasson, S. 2007. A cost-effectiveness analysis of alcohol prevention targeting licensed premises. *Eur.J Public Health*, 17, 618-623.

Mathematica Policy Research, I. 1990, *The savings in medicaid costs for newborns and their mothers from prenatal participation in the WIC program*, U.S. Department of Agriculture, Foor and Nutrition Service, Alexandria, VA.

Mayer, J.P., Hawkins, B., & Todd, R. 1990. A randomized evaluation of smoking cessation interventions for pregnant women at a WIC clinic. *Am.J.Public Health*, 80, 76-78.

McBride, N., Midford, R., Farringdon, F., & Phillips, M. 2000. Early results from a school alcohol harm minimization study: the School Health and Alcohol Harm Reduction Project. *Addiction*, 95, 1021-1042.

McBride, N., Farringdon, F., Midford, R., Meuleners, L., & Phillips, M. 2003. Early unsupervised drinking--reducing the risks. The School Health and Alcohol Harm Reduction Project. *Drug Alcohol Rev.*, 22, 263-276.

McBride, N., Farringdon, F., Midford, R., Meuleners, L., & Phillips, M. 2004. Harm minimization in school drug education: final results of the School Health and Alcohol Harm Reduction Project (SHAHRP). *Addiction*, 99, 278-291.

McDowell, I., Mothersill, K., Rosser, W., & Hartman, R. 1985. A randomized trial of three approaches to smoking cessation. *Can.Fam.Physician*, 31, 845-851.

McGhan, W.F. & Smith, M.D. 1996. Pharmacoeconomic analysis of smoking-cessation interventions. *Am J Health Syst Pharm.*, 53, 45-52.

McKellar, G., Morrison, E., McEntegart, A., Hampson, R., Tierney, A., Mackle, G., Scoular, J., Scott, J.A., & Capell, H.A. 2007. A pilot study of a Mediterranean-type diet intervention in female patients with rheumatoid arthritis living in areas of social deprivation in Glasgow. *Ann.Rheum.Dis*, 66, 1239-1243.

Metcoff, J., Costiloe, P., Crosby, W.M., Dutta, S., Sandstead, H.H., Milne, D., Bodwell, C.E., & Majors, S.H. 1985. Effect of Food Supplementation (Wic) During Pregnancy on Birth-Weight. *American Journal of Clinical Nutrition*, 41, 933-947.

Milch, C.E., Edmunson, J.M., Beshansky, J.R., Griffith, J.L., & Selker, H.P. 2004. Smoking cessation in primary care: a clinical effectiveness trial of two simple interventions. *Prev Med*, 38, 284-294.

Molyneux, A., Lewis, S., Leivers, U., Anderton, A., Antoniak, M., Brackenridge, A., Nilsson, F., McNeill, A., West, R., Moxham, J., & Britton, J. 2003. Clinical trial comparing nicotine replacement therapy (NRT) plus brief counselling, brief counselling alone, and minimal intervention on smoking cessation in hospital inpatients. *Thorax*, 58, 484-488.

Moore, B. L. An investigation of nicotine gum in a smoking cessation program for a respiratory care outer population. Dissertation Abstracts International V47 SECB-PP2626 (University Microfilms International No. 8619925). 1986.
Ref Type: Abstract

Mori, T. A clinical trial of nicotine chewing gum for smoking cessation [abstract 428]. 8thWorld Conference on Tobacco or Health; Buenos Aires, Argentina . 1992.
Ref Type: Generic

Mortimer, D. & Segal, L. 2005. Economic evaluation of interventions for problem drinking and alcohol dependence: cost per QALY estimates. *Alcohol Alcohol*, 40, 549-555.

Muller, P., Abelin, T., Ehrsam, R., Imhof, P., Howald, H., & Mauli, D. 1990. The use of transdermal nicotine in smoking cessation. *Lung*, 168 Suppl, 445-453.

Nagle, A.L., Hensley, M.J., Schofield, M.J., & Koschel, A.J. 2005. A randomised controlled trial to evaluate the efficacy of a nurse-provided intervention for hospitalised smokers. *Aust.N.Z.J.Public Health*, 29, 285-291.

Nebot-Adell, M., Soler-Vila, M., Martin-Cantera, C., Birules-Pons, M., Oller-Colom, M., Sala-Carbonell, E., & Cabezas-Pena, C. 1989. [Effectiveness of the physician's advice to quit smoking: evaluation of the impact a year after the fact]. *Rev.Clin.Esp.*, 184, 201-205.

Nebot, M. & Cabezas, C. 1992. Does nurse counseling or offer of nicotine gum improve the effectiveness of physician smoking-cessation advice? *Fam.Pract.Res.J.*, 12, 263-270.

Nohlert, E., Tegelberg, A., Tillgren, P., Johansson, P., Rosenblad, A., & Helgason, A.R. 2009. Comparison of a high and a low intensity smoking cessation intervention in a dentistry setting in Sweden: a randomized trial. *BMC Public Health*, 9, 121.

Nordlund, S. Effekten av lørdagsstengningen ved Vinmonopolets butikker (Effects of Saturday closing of the wine/liquor monopoly outlets). Alkoholpolitik-Tidsskrift for nordisk alkoholforskning 1, 221-229. 1984.
Ref Type: Abstract

Norstrom, T. & Skog, O.J. 2003. Saturday opening of alcohol retail shops in Sweden: An impact analysis. *Journal of Studies on Alcohol*, 64, 393-401.

O'Connor, A.M., Davies, B.L., Dulberg, C.S., Buhler, P.L., Nadon, C., McBride, B.H., & Benzie, R.J. 1992. Effectiveness of a pregnancy smoking cessation program. *J.Obstet.Gynecol.Neonatal Nurs.*, 21, 385-392.

Ockene, J., Kristeller, J.L., Goldberg, R., Ockene, I., Merriam, P., Barrett, S., Pekow, P., Hosmer, D., & Gianelly, R. 1992. Smoking cessation and severity of disease: the Coronary Artery Smoking Intervention Study. *Health Psychol.*, 11, 119-126.

Ockene, J.K., Kristeller, J., Goldberg, R., Amick, T.L., Pekow, P.S., Hosmer, D., Quirk, M., & Kalan, K. 1991. Increasing the efficacy of physician-delivered smoking interventions: a randomized clinical trial. *J Gen Intern.Med*, 6, 1-8.

Ockene, J.K., Kristeller, J., Goldberg, R., Amick, T.L., Pekow, P.S., Hosmer, D., Quirk, M., & Kalan, K. 1991. Increasing the efficacy of physician-delivered smoking interventions: a randomized clinical trial. *J.Gen.Intern.Med.*, 6, 1-8.

Ockene, J.K., Kristeller, J., Pbert, L., Hebert, J.R., Luippold, R., Goldberg, R.J., Landon, J., & Kalan, K. 1994. The physician-delivered smoking intervention project: can short-term interventions produce long-term effects for a general outpatient population? *Health Psychol.*, 13, 278-281.

Ockene, J.K., Adams, A., Hurley, T.G., Wheeler, E.V., & Hebert, J.R. 1999. Brief physician- and nurse practitioner-delivered counseling for high-risk drinkers: does it work? *Arch.Intern.Med*, 159, 2198-2205.

Orleans, C.T., Schoenbach, V.J., Wagner, E.H., Quade, D., Salmon, M.A., Pearson, D.C., Fiedler, J., Porter, C.Q., & Kaplan, B.H. 1991. Self-help quit smoking interventions: effects of self-help materials, social support instructions, and telephone counseling. *J Consult Clin Psychol.*, 59, 439-448.

Orleans, C.T., Schoenbach, V.J., Wagner, E.H., Quade, D., Salmon, M.A., Pearson, D.C., Fiedler, J., Porter, C.Q., & Kaplan, B.H. 1991. Self-help quit smoking interventions: effects of self-help materials, social support instructions, and telephone counseling. *J.Consult Clin.Psychol.*, 59, 439-448.

Ossip-Klein, D.J., Giovino, G.A., Megahed, N., Black, P.M., Emont, S.L., Stiggins, J., Shulman, E., & Moore, L. 1991. Effects of a smoker's hotline: results of a 10-county self-help trial. *J.Consult Clin.Psychol.*, 59, 325-332.

Oster, G., Huse, D.M., Delea, T.E., & Colditz, G.A. 1986. Cost-effectiveness of nicotine gum as an adjunct to physician's advice against cigarette smoking. *JAMA*, 256, 1315-1318.

Owens, C. S. J. The Roy Castle Fag Ends Stop Smoking Servuce: A successful Client-led Approach to Smoking Cessation. Journal of Smoking Cessation 1, 1-6. 2006.
Ref Type: Abstract

Page, A.R., Walters, D.J., Schlegel, R.P., & Best, J.A. 1986. Smoking cessation in family practice: the effects of advice and nicotine chewing gum prescription. *Addict.Behav.*, 11, 443-446.

Pallonen, U.E., Leskinen, L., Prochaska, J.O., Willey, C.J., Kaariainen, R., & Salonen, J.T. 1994. A 2-year self-help smoking cessation manual intervention among middle-aged Finnish men: an application of the transtheoretical model. *Prev.Med.*, 23, 507-514.

Panjari, M., Bell, R., Bishop, S., Astbury, J., Rice, G., & Doery, J. 1999. A randomized controlled trial of a smoking cessation intervention during pregnancy. *Aust.N.Z.J.Obstet.Gynaecol.*, 39, 312-317.

Paoletti, P., Fornai, E., Maggiorelli, F., Puntoni, R., Viegi, G., Carrozzi, L., Corlando, A., Gustavsson, G., Sawe, U., & Giuntini, C. 1996. Importance of baseline cotinine plasma values in smoking cessation: results from a double-blind study with nicotine patch. *Eur.Respir.J.*, 9, 643-651.

Pederson, L.L., Wood, T., & Lefcoe, N.M. 1983. Use of a self-help smoking cessation manual as an adjunct to advice from a respiratory specialist. *Int.J.Addict.*, 18, 777-782.

Pelletier, J.G. & Moisan, J.T. 1998. [Smoking cessation for hospitalized patients: a quasi-experimental study in Quebec]. *Can.J.Public Health*, 89, 264-269.

Perng, R.P., Hsieh, W.C., Chen, Y.M., Lu, C.C., & Chiang, S.J. 1998. Randomized, double-blind, placebo-controlled study of transdermal nicotine patch for smoking cessation. *J.Formos.Med.Assoc.*, 97, 547-551.

Peterson L, R. A. P. T. K. J. H. J. Smoking reduction during pregnancy by a program of self-help and clinical support. Obstetrics & Gynecology 79, 924-930. 1992.
Ref Type: Abstract

Petrella, R.J., Koval, J.J., Cunningham, D.A., & Paterson, D.H. 2003. Can primary care doctors prescribe exercise to improve fitness? The Step Test Exercise Prescription (STEP) project.  *American Journal of Preventive Medicine*, 24, 316-322.

Porter, A.M. & McCullough, D.M. 1972. Counselling against cigarette smoking. A controlled study from a general practice. *Practitioner*, 209, 686-689.

Postma, M.J., Londeman, J., Veenstra, M., de Walle, H.E., & de Jong-van den Berg LT 2002. Cost-effectiveness of periconceptional supplementation of folic acid. *Pharm.World Sci.*, 24, 8-11.

Pritchard, J.E., Nowson, C.A., & Wark, J.D. 1997. A worksite program for overweight middle-aged men achieves lesser weight loss with exercise than with dietary change. *Journal of the American Dietetic Association*, 97, 37-42.

Prochaska, J.O., DiClemente, C.C., Velicer, W.F., & Rossi, J.S. 1993. Standardized, individualized, interactive, and personalized self-help programs for smoking cessation. *Health Psychol.*, 12, 399-405.

Prochaska, J.O., Velicer, W.F., Fava, J.L., Rossi, J.S., & Tsoh, J.Y. 2001. Evaluating a population-based recruitment approach and a stage-based expert system intervention for smoking cessation. *Addict.Behav*, 26, 583-602.

Prue, D.M., Davis, C.J., Martin, J.E., & Moss, R.A. 1983. An investigation of a minimal contact brand fading program for smoking treatment. *Addict.Behav.*, 8, 307-310.

Purath, J. M. A. M. M. G. &. W. J. A brief intervention to increase physical activity in sedentary working women. Can J Nurs Res 36, 76-91. 2004.
Ref Type: Abstract

Puska, P., Bjorkqvist, S., & Koskela, K. 1979. Nicotine-containing chewing gum in smoking cessation: a double blind trial with half year follow-up. *Addict.Behav*, 4, 141-146.

Rasmussen, S.R., Thomsen, J.L., Kilsmark, J., Hvenegaard, A., Engberg, M., Lauritzen, T., & Sogaard, J. 2007. Preventive health screenings and health consultations in primary care increase life expectancy without increasing costs. *Scand.J Public Health*, 35, 365-372.

Reading, A.E., Campbell, S., Cox, D.N., & Sledmere, C.M. 1982. Health beliefs and health care behaviour in pregnancy. *Psychol.Med.*, 12, 379-383.

Research Committee of the British Thoracic Society. Comparison of four methods of smoking withdrawal in patients with smoking related diseases. Report by a subcommittee of the Research Committee of the British Thoracic Society. British Medical Journal 286 , 595-597. 1983.
Ref Type: Generic

Resnicow, K., Vaughan, R., Futterman, R., Weston, R.E., Royce, J., Parms, C., Hearn, M.D., Smith, M., Freeman, H.P., & Orlandi, M.A. 1997. A self-help smoking cessation program for inner-city African Americans: results from the Harlem Health Connection Project. *Health Educ.Behav.*, 24, 201-217.

Rice, V.H., Fox, D.H., Lepczyk, M., Sieggreen, M., Mullin, M., Jarosz, P., & Templin, T. 1994. A comparison of nursing interventions for smoking cessation in adults with cardiovascular health problems. *Heart Lung*, 23, 473-486.

Rice, V.H. & Stead, L.F. 2004. Nursing interventions for smoking cessation. *Cochrane Database Syst.Rev.* CD001188.

Richmond, R., Heather, N., Wodak, A., Kehoe, L., & Webster, I. 1995. Controlled evaluation of a general practice-based brief intervention for excessive drinking. *Addiction*, 90, 119-132.

Rimer, B.K., Orleans, C.T., Fleisher, L., Cristinzio, S., Resch, N., Telepchak, J., & Keintz, M.K. 1994. Does tailoring matter? The impact of a tailored guide on ratings and short-term smoking-related outcomes for older smokers. *Health Educ.Res*, 9, 69-84.

Roberts, C. S. C. C. J. Quit and win Wales: an evaluation of the 1990 pilot contest. Tobacco Control 2, 114-119. 1993.
Ref Type: Abstract

Romelsjo, A., Andersson, L., Barrner, H., Borg, S., Granstrand, C., Hultman, O., Hassler, A., Kallqvist, A., Magnusson, P., Morgell, R., & . 1989. A randomized study of secondary prevention of early stage problem drinkers in primary health care. *Br.J Addict.*, 84, 1319-1327.

Roto, P. Nicotine gum and withdrawal from smoking. Suomen Laakarllehtl 36, 3445-3448. 1987.
Ref Type: Generic

Rush, D., Horvitz, D.G., Seaver, W.B., Alvir, J.M., Garbowski, G.C., Leighton, J., Sloan, N.L., Johnson, S.S., Kulka, R.A., & Shanklin, D.S. 1988. The National Wic Evaluation - Evaluation of the Special Supplemental Food Program for Women, Infants, and Children - Background and Introduction. *American Journal of Clinical Nutrition*, 48, 389-393.

Russell, M.A., Wilson, C., Taylor, C., & Baker, C.D. 1979. Effect of general practitioners' advice against smoking. *Br.Med.J.*, 2, 231-235.

Russell, M.A., Merriman, R., Stapleton, J., & Taylor, W. 1983. Effect of nicotine chewing gum as an adjunct to general practitioner's advice against smoking. *Br.Med.J.(Clin.Res.Ed)*, 287, 1782-1785.

Russell, R.M., Rosenberg, I.H., Wilson, P.D., Iber, F.L., Oaks, E.B., Giovetti, A.C., Otradovec, C.L., Karwoski, P.A., & Press, A.W. 1983. Increased urinary excretion and prolonged turnover time of folic acid during ethanol ingestion. *Am.J.Clin.Nutr.*, 38, 64-70.

Saffer, H. & Dave, D. 2006. Alcohol advertising and alcohol consumption by adolescents. *Health Econ.*, 15, 617-637.

Salvador Llivina T. Treatment of smoking: efficacy of the use of nicotine chewing gum. Double-blind study. Medicina Clinica Barcelona 90, 646-650. 1988.
Ref Type: Generic

Saunders, J. Successful early intervention for harmful alcohol consumption: Results from the WHO randomised control-ed trial. Proceedings of the Autumn School for Studies in Alcohol and Drugs, 183-192. 1991.
Ref Type: Abstract

Schillinger, J.A., Kissinger, P., Calvet, H., Whittington, W.L., Ransom, R.L., Sternberg, M.R., Berman, S.M., Kent, C.K., Martin, D.H., Oh, M.K., Handsfield, H.H., Bolan, G., Markowitz, L.E., & Fortenberry, J.D. 2003. Patient-delivered partner treatment with azithromycin to prevent repeated Chlamydia trachomatis infection among women: a randomized, controlled trial. *Sex Transm.Dis*, 30, 49-56.

Schneider, N.G., Jarvik, M.E., Forsythe, A.B., Read, L.L., Elliott, M.L., & Schweiger, A. 1983. Nicotine gum in smoking cessation: a placebo-controlled, double-blind trial. *Addict.Behav*, 8, 253-261.

Schneider, N.G. & Jarvik, M.E. 1985. Nicotine gum vs. placebo gum: comparisons of withdrawal symptoms and success rates. *NIDA Res.Monogr*, 53, 83-101.

Schoenbach, V.J., Orleans, C.T., Wagner, E.H., Quade, D., Salmon, M.A., & Porter, C.Q. 1992. Characteristics of smokers who enroll and quit in self-help programs. *Health Education Research*, 7, 369-380.

Schofield, P.E., Hill, D.J., Johnston, C.I., & Streeton, J.A. 1999. The effectiveness of a directly mailed smoking cessation intervention to Australian discharged hospital patients. *Prev.Med.*, 29, 527-534.

Schonlau, M., Scribner, R., Farley, T.A., Theall, K.P., Bluthenthal, R.N., Scott, M., & Cohen, D.A. 2008. Alcohol outlet density and alcohol consumption in Los Angeles county and southern Louisiana. *Geospatial Health*, 3, 91-101.

Schorling, J.B., Roach, J., Siegel, M., Baturka, N., Hunt, D.E., Guterbock, T.M., & Stewart, H.L. 1997. A trial of church-based smoking cessation interventions for rural African Americans. *Prev Med*, 26, 92-101.

Schramm, W.F. 1985. Wic Prenatal Participation and Its Relationship to Newborn Medicaid Costs in Missouri - A Cost-Benefit Analysis. *American Journal of Public Health*, 75, 851-857.

Schramm, W.F. 1986. Prenatal Participation in Wic Related to Medicaid Costs for Missouri Newborns - 1982 Update. *Public Health Reports*, 101, 607-615.

Schuit, A.J., Wendel-Vos, G.C., Verschuren, W.M., Ronckers, E.T., Ament, A., van, A.P., van, R.J., & Ruland, E.C. 2006. Effect of 5-year community intervention Hartslag Limburg on cardiovascular risk factors. *Am.J Prev Med*, 30, 237-242.

Scott, E. & Anderson, P. 1991. Randomized controlled trial of general practitioner intervention in women with excessive alcohol consumption. *Drug Alcohol Rev*, 10, 313-321.

Secker-Walker, R.H., Solomon, L.J., Flynn, B.S., Skelly, J.M., Lepage, S.S., Goodwin, G.D., & Mead, P.B. 1994. Individualized smoking cessation counseling during prenatal and early postnatal care. *Am.J.Obstet.Gynecol.*, 171, 1347-1355.

Senft, R.A., Polen, M.R., Freeborn, D.K., & Hollis, J.F. 1997. Brief intervention in a primary care setting for hazardous drinkers. *Am.J Prev Med*, 13, 464-470.

Severson, H.H., Andrews, J.A., Lichtenstein, E., Gordon, J.S., & Barckley, M.F. 1998. Using the hygiene visit to deliver a tobacco cessation program: results of a randomized clinical trial. *J Am Dent Assoc*, 129, 993-999.

Sexton, M. & Hebel, J.R. 1984. A clinical trial of change in maternal smoking and its effect on birth weight. *JAMA*, 251, 911-915.

Silagy, C., Lancaster, T., Stead, L., Mant, D., & Fowler, G. 2004. Nicotine replacement therapy for smoking cessation. *Cochrane Database Syst.Rev.* CD000146.

Sinclair, H.K., Bond, C.M., Lennox, A.S., Silcock, J., Winfield, A.J., & Donnan, P.T. 1998. Training pharmacists and pharmacy assistants in the stage-of-change model of smoking cessation: a randomised controlled trial in Scotland. *Tob.Control*, 7, 253-261.

Slama, K. Effectiveness of minimal intervention by general practitioners with their smoking patients: a randomised controlled trial in France. 4 , 162-169. 1995.
Ref Type: Generic

Slama, K., Redman, S., Perkins, J., Reid, A.L., & Sanson-Fisher, R.W. 1990. The effectiveness of two smoking cessation programmes for use in general practice: a randomised clinical trial. *BMJ*, 300, 1707-1709.

Smith, B.J., Bauman, A.E., Bull, F.C., Booth, M.L., & Harris, M.F. 2000. Promoting physical activity in general practice: a controlled trial of written advice and information materials.  *British Journal of Sports Medicine*, 34, 262-267.

Smith, M.D., McGhan, W.F., & Lauger, G. 1995. Pharmacist counseling and outcomes of smoking cessation. *Am Pharm.*, NS35, 20-29.

Solberg, L.I., Maciosek, M.V., & Edwards, N.M. 2008. Primary care intervention to reduce alcohol misuse ranking its health impact and cost effectiveness. *Am.J Prev Med*, 34, 143-152.

Solomon, L.J., Secker-Walker, R.H., Flynn, B.S., Skelly, J.M., & Capeless, E.L. 2000. Proactive telephone peer support to help pregnant women stop smoking. *Tob Control*, 9 Suppl 3, III72-III74.

Solomon, L.J., Secker-Walker, R.H., Flynn, B.S., Skelly, J.M., & Capeless, E.L. 2000. Proactive telephone peer support to help pregnant women stop smoking. *Tob.Control*, 9 Suppl 3, III72-III74.

Sonderskov, J., Olsen, J., Sabroe, S., Meillier, L., & Overvad, K. 1997. Nicotine patches in smoking cessation: a randomized trial among over-the-counter customers in Denmark. *Am.J.Epidemiol.*, 145, 309-318.

Stead, L., Bergson, G., & Lancaster, T. Physician advice for smoking cessation (Review). Cochrane Database of Systematic Reviews , CD000165. 2008.
Ref Type: Generic

Stevens, W., Thorogood, M., & Kayikki, S. 2002. Cost-effectiveness of a community anti-smoking campaign targeted at a high risk group in London. *Health Promot.Int*, 17, 43-50.

Stewart, P.J. & Rosser, W.W. 1982. The impact of routine advice on smoking cessation from family physicians. *Can.Med.Assoc.J.*, 126, 1051-1054.

Stockbauer, J.W. 1986. Evaluation of the Missouri Wic Program - Prenatal Components. *Journal of the American Dietetic Association*, 86, 61-67.

Stockbauer, J.W. 1987. Wic Prenatal Participation and Its Relation to Pregnancy Outcomes in Missouri - A 2Nd Look. *American Journal of Public Health*, 77, 813-818.

Stoddard, A.M., Palombo, R., Troped, P.J., Sorensen, G., & Will, J.C. 2004. Cardiovascular disease risk reduction: the Massachusetts WISEWOMAN project. *J Womens Health (Larchmt.)*, 13, 539-546.

Stotts, A.L., DiClemente, C.C., & Dolan-Mullen, P. 2002. One-to-one: a motivational intervention for resistant pregnant smokers. *Addict.Behav.*, 27, 275-292.

Strecher, V.R.B. 1993. *Freedom from smoking* New York, American Lung Association.

Strecher, V.J., Bishop, K.R., Bernhardt, J., Thorp, J.M., Cheuvront, B., & Potts, P. 2000. Quit for keeps: tailored smoking cessation guides for pregnancy and beyond. *Tob.Control*, 9 Suppl 3, III78-III79.

Susan Battersby, S. The cost-effectiveness of breastfeeding peer support. British Journal of Midwifery 12, 201-205. 2004.
Ref Type: Abstract

Sutton, S. & Hallett, R. 1987. Randomized trial of brief individual treatment for smoking using nicotine chewing gum in a workplace setting. *Am.J.Public Health*, 77, 1210-1211.

Sutton, S. & Hallett, R. 1988. Smoking intervention in the workplace using videotapes and nicotine chewing gum. *Prev.Med.*, 17, 48-59.

Swinburn, B.A., Walter, L.G., Arroll, B., Tilyard, M.W., & Russell, D.G. 1998. The green prescription study: A randomized controlled trial of written exercise advice provided by general practitioners. *American Journal of Public Health*, 88, 288-291.

Tappin, D.M., Lumsden, M.A., McIntyre, D., Mckay, C., Gilmour, W.H., Webber, R., Cowan, S., Crawford, F., & Currie, F. 2000. A pilot study to establish a randomized trial methodology to test the efficacy of a behavioural intervention. *Health Educ.Res.*, 15, 491-502.

Taskanen, A. Change in risk factors for coronary heart disease during 10 years of a community intervention program (North Karelia project). Br Med J 287, 1840-1844. 1983.
Ref Type: Abstract

Taylor, C.B., Houston-Miller, N., Killen, J.D., & DeBusk, R.F. 1990. Smoking cessation after acute myocardial infarction: effects of a nurse-managed intervention. *Ann.Intern.Med*, 113, 118-123.

Thompson, R.S., Michnich, M.E., Friedlander, L., Gilson, B., Grothaus, L.C., & Storer, B. 1988. Effectiveness of smoking cessation interventions integrated into primary care practice. *Med.Care*, 26, 62-76.

Thornton L. Smoking and pregnancy: feasibility and effectiveness of a smoking intervention programme among pregnant women. Dublin:Dept of Public Health . 1997.
Ref Type: Abstract

Tillgren, P., Haglund, B.J., Gilljam, H., & Holm, L.E. 1992. A tobacco quit and win model in the Stockholm cancer prevention programme. *Eur J Cancer Prev*, 1, 361-366.

Tillgren, P., Eriksson, L., Guldbrandsson, K., & Spiik, M. 2000. Impact of direct mail as a method to recruit smoking mothers into a "quit and win" contest. *J Health Commun.*, 5, 293-303.

Tonnesen, P., Fryd, V., Hansen, M., Helsted, J., Gunnersen, A.B., Forchammer, H., & Stockner, M. 1988. Two and four mg nicotine chewing gum and group counselling in smoking cessation: an open, randomized, controlled trial with a 22 month follow-up. *Addict.Behav.*, 13, 17-27.

Tonnesen, P., Norregaard, J., Simonsen, K., & Sawe, U. 1991. A double-blind trial of a 16-hour transdermal nicotine patch in smoking cessation. *N Engl J Med*, 325, 311-315.

Tonnesen, P., Norregaard, J., Simonsen, K., & Sawe, U. 1991. A double-blind trial of a 16-hour transdermal nicotine patch in smoking cessation. *N.Engl.J.Med.*, 325, 311-315.

Tonnesen, P., Mikkelsen, K., Markholst, C., Ibsen, A., Bendixen, M., Pedersen, L., Fuursted, R., Hansen, L.H., Stensgaard, H., Schiotz, R., Petersen, T., Breman, L., Clementsen, P., & Evald, T. 1996. Nurse-conducted smoking cessation with minimal intervention in a lung clinic: a randomized controlled study. *Eur.Respir.J.*, 9, 2351-2355.

Tonnesen, P., Paoletti, P., Gustavsson, G., Russell, M.A., Saracci, R., Gulsvik, A., Rijcken, B., & Sawe, U. 1999. Higher dosage nicotine patches increase one-year smoking cessation rates: results from the European CEASE trial. Collaborative European Anti-Smoking Evaluation. European Respiratory Society. *Eur.Respir.J.*, 13, 238-246.

Tosteson, A.N., Weinstein, M.C., Hunink, M.G., Mittleman, M.A., Williams, L.W., Goldman, P.A., & Goldman, L. 1997. Cost-effectiveness of populationwide educational approaches to reduce serum cholesterol levels. *Circulation*, 95, 24-30.

Turner, L.R., Morera, O.F., Johnson, T.P., Crittenden, K.S., Freels, S., Parsons, J., Flay, B., & Warnecke, R.B. 2001. Examining the effectiveness of a community-based self-help program to increase women's readiness for smoking cessation. *Am J Community Psychol.*, 29, 465-491.

Valbo, A. & Schioldborg, P. 1991. Smoking cessation in pregnancy. Mode of intervention and effect. *Acta Obstet.Gynecol.Scand.*, 70, 309-313.

Vetter, N.J. & Ford, D. 1990. Smoking prevention among people aged 60 and over: a randomized controlled trial. *Age Ageing*, 19, 164-168.

Vidrine, D.J., Arduino, R.C., Lazev, A.B., & Gritz, E.R. 2006. A randomized trial of a proactive cellular telephone intervention for smokers living with HIV/AIDS. *AIDS*, 20, 253-260.

Viswesvaran, C. & Schmidt, F.L. 1992. A meta-analytic comparison of the effectiveness of smoking cessation methods. *J Appl.Psychol.*, 77, 554-561.

Wadden, T.A., Sternberg, J.A., Letizia, K.A., Stunkard, A.J., & Foster, G.D. 1989. Treatment of Obesity by Very Low Calorie Diet, Behavior-Therapy, and Their Combination - A 5-Year Perspective. *International Journal of Obesity*, 13, 39-46.

Wagenaar, A.C., Salois, M.J., & Komro, K.A. 2009. Effects of beverage alcohol price and tax levels on drinking: a meta-analysis of 1003 estimates from 112 studies. *Addiction*, 104, 179-190.

Wallace, P., Cutler, S., & Haines, A. 1988. Randomised controlled trial of general practitioner intervention in patients with excessive alcohol consumption. *BMJ*, 297, 663-668.

Walsh, R.A., Redman, S., Brinsmead, M.W., Byrne, J.M., & Melmeth, A. 1997. A smoking cessation program at a public antenatal clinic. *Am.J.Public Health*, 87, 1201-1204.

Warner, K.E. 1997. Cost effectiveness of smoking-cessation therapies. Interpretation of the evidence-and implications for coverage. *Pharmacoeconomics.*, 11, 538-549.

Werch, C.E., Carlson, J.M., Pappas, D.M., Edgemon, P., & DiClemente, C.C. 2000. Effects of a brief alcohol preventive intervention for youth attending school sports physical examinations. *Subst.Use.Misuse.*, 35, 421-432.

Werch, C.E., Carlson, J.M., Owen, D.M., DiClemente, C.C., & Carbonari, J.P. 2001. Effects of a stage-based alcohol preventive intervention for inner-city youth. *J Drug Educ.*, 31, 123-138.

Werch, C.E., Owen, D.M., Carlson, J.M., DiClemente, C.C., Edgemon, P., & Moore, M. 2003. One-year follow-up results of the STARS for Families alcohol prevention program. *Health Educ.Res*, 18, 74-87.

Wilson, D.H., Wakefield, M.A., Steven, I.D., Rohrsheim, R.A., Esterman, A.J., & Graham, N.M. 1990. "Sick of Smoking": evaluation of a targeted minimal smoking cessation intervention in general practice. *Med.J.Aust.*, 152, 518-521.

Windsor, R.A., Cutter, G., Morris, J., Reese, Y., Manzella, B., Bartlett, E.E., Samuelson, C., & Spanos, D. 1985. The effectiveness of smoking cessation methods for smokers in public health maternity clinics: a randomized trial. *Am.J.Public Health*, 75, 1389-1392.

Windsor, R.A., Lowe, J.B., Perkins, L.L., Smith-Yoder, D., Artz, L., Crawford, M., Amburgy, K., & Boyd, N.R., Jr. 1993. Health education for pregnant smokers: its behavioral impact and cost benefit. *Am.J.Public Health*, 83, 201-206.

Windsor, R.A., Woodby, L.L., Miller, T.M., Hardin, J.M., Crawford, M.A., & DiClemente, C.C. 2000. Effectiveness of Agency for Health Care Policy and Research clinical practice guideline and patient education methods for pregnant smokers in medicaid maternity care. *Am.J.Obstet.Gynecol.*, 182, 68-75.

Wisborg, K., Henriksen, T.B., Jespersen, L.B., & Secher, N.J. 2000. Nicotine patches for pregnant smokers: a randomized controlled study. *Obstet.Gynecol.*, 96, 967-971.

Wonderling, D., McDermott, C., Buxton, M., Kinmonth, A.L., Pyke, S., Thompson, S., & Wood, D. 1996. Costs and cost effectiveness of cardiovascular screening and intervention: the British family heart study. *BMJ*, 312, 1269-1273.

Wonderling, D., Langham, S., Buxton, M., Normand, C., & McDermott, C. 1996. What can be concluded from the Oxcheck and British family heart studies: commentary on cost effectiveness analyses. *BMJ*, 312, 1274-1278.

Wood, P.D., Stefanick, M.L., Williams, P.T., & Haskell, W.L. 1991. The Effects on Plasma-Lipoproteins of A Prudent Weight-Reducing Diet, with Or Without Exercise, in Overweight Men and Women. *New England Journal of Medicine*, 325, 461-466.

Wrieden, W.L., Anderson, A.S., Longbottom, P.J., Valentine, K., Stead, M., Caraher, M., Lang, T., Gray, B., & Dowler, E. 2007. The impact of a community-based food skills intervention on cooking confidence, food preparation methods and dietary choices - an exploratory trial. *Public Health Nutr.*, 10, 203-211.

Xie, X., Mann, R.E., & Smart, R.G. 2000. The direct and indirect relationships between alcohol prevention measures and alcoholic liver cirrhosis mortality. *Journal of Studies on Alcohol*, 61, 499-506.

Zelman, D. C. Treatments for cigarette smoking: The contribution of counselling and nicotine replacement strategies (Doctoral Dissertation). University of Wisconsin, Madison (Dissertation Abstracts International V50 PP5899). 2012.
Ref Type: Abstract

Zhu, S.H., Stretch, V., Balabanis, M., Rosbrook, B., Sadler, G., & Pierce, J.P. 1996. Telephone counseling for smoking cessation: effects of single-session and multiple-session interventions. *J Consult Clin Psychol.*, 64, 202-211.
