# Supplementary material for: What do cost-effective health behaviour-change interventions contain? A comparison of six domains
Source: PLoS One. 2019 Apr 17;14(4):e0213983. doi: 10.1371/journal.pone.0213983 (PMC6469762; doi:10.1371/journal.pone.0213983)
Supplement: S1 Table — (DOCX) [file pone.0213983.s003.docx]

| ***Health-behaviour*** | ***Present in up to 25% of interventions*** | ***Present in 25% to 50% of interventions*** | ***Present in 50% to 75% of interventions*** | ***Present in 75-100% of interventions*** |
| --- | --- | --- | --- | --- |
| **Smoking** | Restructuring the physical environment (BCT 30)  Material incentive (behaviour) (BCT 60)  Avoidance/reducing exposure to cues for the behaviour (BCT 32)  Self-monitoring of outcome(s) of behaviour (BCT 11)  Monitoring outcome(s) of behaviour by others without feedback (BCT 13)  Distraction (BCT 33)  Incompatible beliefs (BCT 80)  Identity associated with changed behaviour (BCT 81)  Anticipated regret (BCT 87)  Review behaviour goal (BCT 69)  Self-reward (BCT 58)  Non-specific incentive (BCT 63)  Mental rehearsal of successful performance (BCT 41)  Information about others’ approval (BCT 90)  Imaginary punishment (BCT 91)  Non-specific reward (BCT 57)  Social support (practical) (BCT 1)  Reduce prompts/cues (BCT 16)  Punishment (BCT 44)  Future punishment (BCT 59)  Goal setting (behaviour) (BCT 66)  Social support (emotional) (BCT 2)  Framing/reframing (BCT 79)  Information about social and environmental consequences (BCT 84)  Paradoxical instructions (BCT 7)  Social comparison (BCT 89)  Behavioural contract (BCT 71)  Commitment (BCT 72)  Behavioural practice/rehearsal (BCT 23)  Material reward (behaviour) (BCT 54)  Social reward (BCT 56)  Habit reversal (BCT 26)  Feedback on behaviour (BCT 8)  Body changes (BCT 35)  Biofeedback (BCT 14)  Graded tasks (BCT 29)  Behaviour substitution (BCT 25)  Material reward (outcome) (BCT 55)  Salience of consequences (BCT 85)  Pros and cons (BCT 75)  Information about emotional consequences (BCT 83)  Prompts/cues (BCT 15)  Self-monitoring of behaviour (BCT 10)  Demonstration of behaviour (BCT 88) | Adding objects to the environment (BCT 34)  Verbal persuasion about capability (BCT 40)  Reduce negative emotions (BCT 5)  Action planning (BCT 68) | Pharmacological support (BCT 4)  Information about health consequences (BCT 82)  Problem solving (BCT 65)  Persuasive source (BCT 74)  Goal setting (outcome) (BCT 67) | Social support (unspecified) (BCT 3)  Instruction on how to perform a behaviour (BCT 36) |

| ***Health-behaviour*** | ***Present in up to 25% of interventions*** | ***Present in 25% to 50% of interventions*** | ***Present in 50% to 75% of interventions*** | ***Present in 75-100% of interventions*** |
| --- | --- | --- | --- | --- |
| **Diet** | Pharmacological support(BCT 4)  Reduce prompts/cues (BCT 16)  Goal setting (behaviour) (BCT 66)  Social comparison (BCT 89)  Graded tasks (BCT 29)  Action planning (BCT 68)  Verbal persuasion about capability (BCT 40) | Self-monitoring of behaviour (BCT 10)  Social support (unspecified) (BCT 3)  Problem solving (BCT 65)  Information about health consequences (BCT 82) | Adding objects to the environment (BCT 34) | Instructions on how to perform a behaviour (BCT 36)  Body changes (BCT 35) |
| **Sexual Health** | Information about antecedents (BCT 37)  Social reward (BCT 56)  Material reward (outcomes) (BCT 55)  Material reward (behaviour) (BCT 54)  Pros and cons (BCT 75)  Social comparison (BCT 89)  Information about emotional consequences (BCT 83)  Reduce negative emotion (BCT5)  Graded tasks (BCT 29)  Demonstration of behaviour (BCT 88)  Goal setting (behaviour) (BCT 66)  Information about social and environmental consequences (BCT 84) | Behavioural practice/rehearsal (BCT 23)  Behaviour substitution (BCT 25)  Information about health consequences (BCT 82) | Social support (unspecified) (BCT 3)  Biofeedback (BCT 14)  Persuasive source (BCT 74)  Adding objects to the environment (BCT 34)  Verbal persuasion about capability (BCT 40)  Action planning (BCT 68)  Framing/reframing (BCT 79) | Instructions on how to perform a behaviour (BCT 36)  Feedback on behaviour (BCT 8)  Problem solving (BCT 65) |
| **Alcohol** | Self-reward (BCT 58)  Behavioural practice/rehearsal (BCT 23)  Material reward (behaviour) (BCT 54)  Behaviour substitution (BCT 25)  Biofeedback (BCT 14)  Pros and cons (BCT 75)  Social comparison (BCT 89)  Self-incentive (BCT 64)  Information about emotional consequences (BCT 83)  Reduce negative emotions (BCT 5)  Self-monitoring of behaviour (BCT 10)  Behavioural contract (BCT 71)  Graded tasks (BCT 29)  Material incentive (behaviour) (BCT 60)  Review behaviour goal (BCT 69)  Feedback on behaviour (BCT 8)  Demonstration of behaviour (BCT 88)  Goal setting (behaviour) (BCT 66) | Prompts/cues (BCT 15)  Framing/reframing (BCT 79)  Avoidance/reducing exposure to cues for the behaviour (BCT 32)  Information about social and environmental consequences (BCT 84)  Verbal persuasion about capability (BCT 40)  Action planning (BCT 68)  Instruction on how to perform a behaviour (BCT 36) | Restructuring the physical environment (BCT 30)  Problem solving (BCT 65)  Persuasive source (BCT 74)  Information about health consequences (BCT 82)  Social support (Practical) (BCT 1) |  |

| ***Health-behaviour*** | ***Present in up to 25% of interventions*** | ***Present in 25% to 50% of interventions*** | ***Present in 50% to 75% of interventions*** | ***Present in 75-100% of interventions*** |
| --- | --- | --- | --- | --- |
| **Physical Activity** | Remove aversive stimulus (BCT 19)  Satiation (BCT 20)  Restructuring the social environment (BCT 31)  Non-specific incentive (BCT 63)  Social support (practical) (BCT 1)  Framing/reframing (BCT 79)  Behavioural contract (BCT 71)  Biofeedback (BCT 14)  Graded tasks (BCT 29)  Restructuring the physical environment (BCT 30)  Material incentive (behaviour) (BCT 60)  Review behaviour goal (BCT 69)  Non-specific reward (BCT 57)  Information about social and environmental consequences (BCT 84)  Feedback on behaviour (BCT 8)  Pros and cons (BCT 75)  Demonstration of behaviour (BCT 88)  Goal setting (outcome) (BCT 67)  Social comparison (BCT 89)  Verbal persuasion about capability (BCT 40)  Problem solving (BCT 65) | Adding objects to the environment (BCT 34)  Action planning (BCT 68)  Persuasive source (BCT 74) | Social support (unspecified) (BCT 3)  Instructions on how to perform a behaviour (BCT 36)  Goal setting (behaviour) (BCT 66)  Information about health consequences (BCT 82) | Body changes (BCT 35) |
| **Multiple Behaviours** | Material incentive (behaviour) (BCT 60)  Review behaviour goal(s) (BCT 69)  Material reward (behaviour) (BCT 54)  Information about emotional consequences (BCT 83)  Reduce negative emotion (BCT5)  Information about social and environmental consequences (BCT 84)  Behavioural practice/rehearsal (BCT 23)  Behaviour substitution (BCT 25)  Framing/reframing (BCT 79)  Adding objects to the environment (BCT 34) | Biofeedback (BCT 14)  Demonstration of behaviour (BCT 88)  Social comparison (BCT 89) | Self-monitoring of behaviour (BCT 10)  Prompts/cues (BCT 15)  Restructuring the physical environment (BCT 30)  Problem solving (BCT 65)  Persuasive source (BCT 74) | Information about health consequences (BCT 82)  Instructions on how to perform a behaviour (BCT 36)  Goal setting (behaviour) (BCT 66)  Feedback on behaviour (BCT 8)  Social support (unspecified) (BCT 3) |
