# Supplementary material for: What do cost-effective health behaviour-change interventions contain? A comparison of six domains
Source: PLoS One. 2019 Apr 17;14(4):e0213983. doi: 10.1371/journal.pone.0213983 (PMC6469762; doi:10.1371/journal.pone.0213983)
Supplement: S2 Table — (DOCX) [file pone.0213983.s004.docx]

Supplementary Table 2: Broad categorisation of interventions

| Category distinctions | Description |
| --- | --- |
| Intervention intensity | Low: one face-to-face contact or other direct contact lasting up to 5 minutes or any non-specific (impersonal, e.g. through media) contact  Medium: one face-to-face contact or other direct contact lasting more than 5 minutes, or one face-to-face contact or other direct contact lasting up to 5 minutes on more than one occasion  High: any face-to-face contact or other direct contact lasting more than 5 minutes on more than one occasion. |
| Setting | Primary or secondary care  Community  Workplace  Other |
| Mode of delivery | Physician  Health-care professional (included nurses, pharmacists, psychologists, dieticians and other qualified personnel)  Media  Combination  Other/not specified |
| Target level | Individual  Group  Population |
| Supporting material | Self-help (written)  Electronic (e.g. telephone, mobile phone, computer)  Micture  None |
| Use of pharmacological support | It was recorded whether or not pharmacological support was provided in addition to the type of medication (e.g. NRT for smoking cessation) |
| Use of incentives | It was recorded whether or not incentives were provided to participate in the study and continue with the intervention |
| Social marketing | It was recorded whether social-marketing was implemented during the intervention. Social Marketing was defined as the process of using principles and techniques to influence target audience behaviors that will benefit society. |
| Population | General population  Vulnerable population (e.g. pregnant women, individuals at risk of disease, and those from lower socio-economic groups) |
